# Genomic surveillance reveals escalating antimicrobial resistance and plasmid diversity in clinical *Salmonella* 1,4,[5],12:i:- ST34 isolates from Guizhou Province, China

**DOI:** 10.3389/fmicb.2026.1875505

**Published:** 2026-08-28

**Authors:** Ge Zhu, Lv You, Deyu Li, Junhua Wang, Shijun Li, Hui Liu, Xiaoyu Wei

**Affiliations:** 1 The Key Laboratory of Environmental Pollution Monitoring and Disease Control, School of Public Health, Ministry of Education, Guizhou Medical University, Guiyang, Guizhou, China; 2 Laboratory of Bacterial Disease, Experimental Center, Guizhou Provincial Center for Disease Control and Prevention, Guiyang, China; 3 Preparatory College, Guizhou Provincial Center for Disease Control and Prevention, Guiyang, China; 4 Experimental Center, Guizhou Provincial Center for Disease Control and Prevention, Guiyang, China; 5 School of Public Health, The Key Laboratory of Environmental Pollution Monitoring and Disease Control, Guizhou Medical University, Guizhou, China

**Keywords:** molecular epidemiology, plasmids, *Salmonella* 1,4,[5],12:i:-, ST34, whole-genome sequencing

## Abstract

**Introduction:**

*Salmonella* 1,4,[5],12:i:- ST34 has emerged as a significant public health issue due to its association with various antimicrobial resistance genes (ARGs) and transferable plasmids. However, its genomic characteristics and potential influence on public health in Guizhou have not been comprehensively assessed.

**Methods:**

From 2019 to 2023, a 5-year surveillance was conducted in nine cities (prefectures) of Guizhou Province. We integrated phenotypic and genomic analyses of 281 clinical *Salmonella* 1,4,[5],12:i:- ST34 isolates to investigate the prevalence of ARGs and plasmids and to analyze the molecular epidemiology and evolution.

**Results:**

The isolates exhibited resistance to first-line antibiotics, with 22.4% for ciprofloxacin, 11.4% for azithromycin, 18.5% for ceftazidime, and 39.1% for cefotaxime. ARGs showed substantial agreement with phenotypes for tetracycline, macrolides, third-generation cephalosporins (3GCs), carbapenems, and colistin (80.8–100.0% consistency; *Kappa*: 0.50–1.00). Plasmid analysis identified IncQ1 (84.3%) and IncHI2/IncHI2A (26.3%) as the main replicons, with the variety of plasmid replicons increasing from 7 to 21 over the 5 years. ARGs associated with resistance to critically important antibiotics (CIAs) were frequently predicted to be located on plasmid-associated contigs, with significant associations observed between IncHI2/IncHI2A plasmids and ARGs conferring resistance to fluoroquinolones, macrolides, and cephalosporins (*P* < 0.05). Molecular typing divided 281 isolates into 37 cgSTs, with cgST52428 being the most common. Molecular epidemiological analysis revealed that Guizhou isolates primarily clustered together, sharing close genetic ties with those from Sichuan and Guangdong, and exhibited the highest genetic similarity to pork-derived isolates. Phylogenetic analysis revealed clustering of CIA-resistant ARGs and plasmids in Clades 4 and 5, with a significant association between IncHI2/IncHI2A plasmids and CIA-resistant ARGs (χ^2^ = 112.12, *P* < 0.001). Additionally, class 1 integron was associated with higher ARG burdens, while virulence-associated genes were conserved and predominantly chromosome-associated. Gene-content analysis revealed that isolates in Clades 4 and 5 harbored the largest mean gene complements, and cgST52428 isolates also harbored the largest among dominant cgSTs.

**Discussion:**

This study presents a comprehensive genomic profile of *Salmonella* 1,4,[5],12:i:- ST34 in Guizhou, providing essential data for exploring the resistance characteristics and investigating the molecular epidemiology of *Salmonella* 1,4,[5],12:i:-.

## 1 Introduction

*Salmonella enterica* serovar 1,4,[5],12:i:- (*Salmonella* 1,4,[5],12:i:-), a monophasic variant derived from *Salmonella* Typhimurium, has emerged as a result of the loss of phase II flagellar expression ability (Sun et al., 2020). Over the past two decades, *Salmonella* 1,4,[5],12:i:- has experienced a rapid global spread, becoming one of the most prevalent *Salmonella enterica* serotypes associated with human infections (European Food Safety Authority (EFSA) and European Centre for Disease Prevention and Control (ECDC), 2023). The emergence of *Salmonella* 1,4,[5],12:i:-, coupled with its multidrug resistance and association with foodborne disease outbreaks, poses a significant threat to public health (Clark et al., 2020; Elnekave et al., 2018; Wu et al., 2021). Epidemiological studies have shown that *Salmonella* 1,4,[5],12:i:- is among the top five *Salmonella* serotypes responsible for human infections in the United States (Marder Mph et al., 2018) and ranks as the second most common *Salmonella enterica* serotype in the European Union (European Food Safety Authority (EFSA) and European Centre for Disease Prevention and Control (ECDC), 2019). Research in China has shown a steady increase in the isolation rate of *Salmonella* 1,4,[5],12:i:- from human sources since 2011. By 2016, it surpassed *Salmonella* Typhimurium to become the second most prevalent *Salmonella* serotype (Wang Y. et al., 2023). Studies conducted in Guizhou Province between 2013 and 2018 indicated that *Salmonella* 1,4,[5],12:i:- has also become the second most prevalent serotype, following *Salmonella* Enteritidis (Bai et al., 2022; Wei et al., 2023). *Salmonella* 1,4,[5],12:i:- isolates from Guizhou Province were divided into two sequence types (STs): ST19 and ST34. Among these isolates, ST34 accounted for 97.7% (Long et al., 2022).

The most common resistance phenotype in *Salmonella* 1,4,[5],12:i:- ST34 is ASSuT, including ampicillin, streptomycin, sulfonamides, and tetracyclines. Most isolates exhibiting this phenotype harbor corresponding ARGs, such as *bla*_*TEM*−1_, *strA-strB, sul2*, and *tet(B)* (Garcia et al., 2016). Furthermore, according to the World Health Organization (WHO), critically important antibiotics (CIAs) are antimicrobial classes that serve as sole or limited therapies for serious human infections and are used to treat infections caused by bacteria that may be transmitted from non-human sources. Within this category, fluoroquinolones, third-generation cephalosporins (3GCs), and colistin are classified as highest priority critically important antimicrobials (HP-CIAs), whereas macrolides are classified as CIAs (World Health Organization, 2024). Recent studies have increasingly reported the carriage of ARGs conferring resistance to these CIAs on plasmids in *Salmonella* 1,4,[5],12:i:- ST34 (Xie et al., 2022; Zeng et al., 2023; Wang et al., 2023c). These plasmids, along with transposons, integrons, and insertion sequences, constitute mobile genetic elements (MGEs) that facilitate the horizontal transfer of ARGs, posing significant challenges for treating *Salmonella* 1,4,[5],12:i:- infections. A study of human-derived *Salmonella* in Chengdu revealed that *Salmonella* 1,4,[5],12:i- possessed a greater diversity of plasmid replicons than other serotypes did, with nearly all the isolates containing IncQ1 (Xiao et al., 2022). Moreover, other studies have suggested that the acquisition of large multidrug-resistant (MDR) plasmids, such as IncHI2 or IncA/C2, contributes to the successful expansion of *Salmonella* 1,4,[5],12:i:- ST34 and increases resistance to CIAs (Chung The et al., 2023; Zhao et al., 2024). Surveillance data from Guizhou Province between 2013 and 2018 showed that the MDR rate of *Salmonella* 1,4,[5],12:i:- reached 94.2%, with 13.8% of isolates carrying extended-spectrum β-lactamase (*ESBL*) genes such as *bla*_*CTX*−*M*_ (Long et al., 2022). However, research on AMR in *Salmonella* 1,4,[5],12:i:- ST34 from Guizhou has mainly been phenotypic, with limited data on resistance mechanisms to CIAs. The role of plasmid-associated resistance remains unclear, and the genetic context and evolutionary characteristics of *Salmonella* 1,4,[5],12:i:- ST34 in this region are still unknown. This knowledge gap is significant because understanding the resistance mechanisms and molecular epidemiology is crucial for developing effective treatment strategies and combating the spread of antibiotic resistance. Therefore, comprehensive studies on the AMR and molecular epidemiology of *Salmonella* 1,4,[5],12:i:- ST34 in Guizhou Province are urgently needed.

Whole-genome sequencing (WGS) has become an essential technology for investigating bacterial antimicrobial resistance and molecular epidemiology (Cuypers et al., 2023; Li et al., 2022; Wang et al., 2022). Core genome multilocus sequence typing (cgMLST) and core genome single-nucleotide polymorphism (cgSNP) analyses have proven to be highly effective in differentiating outbreak-related isolates from sporadic cases. Specifically, cgMLST provides high-resolution typing by comparing sequences of core genome loci, which allows for precise assessment of genetic relatedness between isolates and offers essential support for epidemiological traceback investigations (Wang et al., 2023b; Zhang et al., 2024). Additionally, cgSNP analysis focuses on single-nucleotide polymorphisms within the core genome to determine the evolutionary relationships among strains of the same bacterial species. This method has demonstrated high discriminatory power in tracing outbreaks of various common *Salmonella* serotypes (Garcia-Soto et al., 2021; Larkin et al., 2022).

This study performed a phenotypic resistance analysis on *Salmonella* 1,4,[5],12:i:- ST34 isolates from Guizhou Province between 2019 and 2023 to evaluate local AMR trends. Using WGS data, we systematically identified ARGs and plasmids to determine their prevalence among local *Salmonella* 1,4,[5],12:i:- isolates and to elucidate the distribution of plasmid-associated ARGs. We further screened for class 1 integrons, insertion sequences, and virulence-associated genes to explore their genetic context and potential relationships with ARGs. Furthermore, cgMLST and cgSNP analyses were performed to characterize the molecular epidemiology and evolutionary relationships of *Salmonella* 1,4,[5],12:i:- in Guizhou, thereby providing a scientific basis for formulating prevention and control strategies for *Salmonella* 1,4,[5],12:i:-.

## 2 Materials and methods

### 2.1 Isolate sources and collection

Between January 2019 and December 2023, diarrheal stool samples were systematically collected from outpatients at 109 sentinel hospitals across nine cities (prefectures) in Guizhou Province, as part of the Chinese Pathogen Identification Network (CPIN) surveillance program. Patients were eligible for inclusion if they presented with (i) three or more loose stools within 24 h accompanied by altered stool consistency (e.g., watery, mucoid), or (ii) fever (≥38 °C) with accompanying symptoms such as headache, chills, or malaise. All samples were from sporadic community-acquired infections. Patients with hospital-acquired infections or a history of hospitalization within the previous 30 days were excluded. Only one isolate per patient was included to avoid duplication.

All recovered *Salmonella* isolates were submitted to the laboratory of Guizhou Provincial Center for Disease Control and Prevention for confirmatory identification. Species identification was performed using the MALDI-TOF MS system MS1,000 (Autobio Diagnostics Co., Ltd., China), and serotyping was conducted by slide agglutination for O and H antigens (SSI, Denmark) in accordance with the White-Kauffmann-Le Minor scheme (Guibourdenche et al., 2010). From these, 284 isolates were subsequently identified as *Salmonella* 1,4,[5],12:i:- using multiplex polymerase chain reaction (mPCR) (Tennant et al., 2010). Bacterial DNA was extracted by the boiling lysis method, and the supernatant was used as a DNA template and stored at −20 °C until use. The multiple PCR cycling conditions were as follows: initial denaturation at 95 °C for 2 min, 30 cycles of denaturation at 95 °C for 30 s, annealing at 58 °C for 30 s, extension at 72 °C for 90 s, and final extension at 72 °C for 10 min (Supplementary Figures S1–S11).

### 2.2 Antimicrobial susceptibility testing

Minimum inhibitory concentrations (MICs) were determined using the broth microdilution method, as outlined by the Clinical and Laboratory Standards Institute (CLSI, 2023) (Supplementary Data S1). To ensure comparability across sampling years, all isolates collected from 2019 to 2023 were stored and subsequently tested in a single batch using the same lot of Customized AST plate CHNENF (Thermo Fisher Scientific, USA). A total of 17 antibiotics from 11 different categories were tested, including quinolones and fluoroquinolones (ciprofloxacin-CIP, nalidixic acid-NA), macrolides (azithromycin-AZM), aminoglycosides (amikacin-AN, streptomycin-STS), penicillin (ampicillin-AM), phenicol (chloramphenicol-C), sulfonamides (trimethoprim-sulfamethoxazole-SXT), tetracyclines (tetracycline-TE, tigecycline-TGC), carbapenems (meropenem-MEM, ertapenem-ETP), cephems (ceftazidime-CAZ, cefotaxime-CTX), lipopeptide (colistin-CL), and β-lactamase inhibitor (ampicillin/sulbactam-SAM, ceftazidime/avibactam-CZA). *Escherichia coli* ATCC 25922 was used as a quality control, and the WHONET 2023 software (https://whonet.org/software.html) was used to analyze the antimicrobial resistance data. Multidrug-resistant (MDR) *Salmonella* is defined as being resistant to at least three different classes of antibiotics, and extensively drug-resistant (XDR) *Salmonella* is defined as remaining susceptible to only one or two classes (Magiorakos et al., 2012).

### 2.3 DNA extraction and whole-genome sequencing

Genomic DNA extraction was performed for *Salmonella* 1,4,[5],12:i:- ST34 isolates using a magnetic bacterial genomic DNA kit (Baiju Technologies, China). The quality and concentration of the extracted bacterial genomic DNA were assessed through electrophoresis on a 1% agarose gel, and quantification was conducted by a Qubit 4 fluorometer (Thermo Scientific, Waltham, USA).

The DNA library construction, circularization, and preparation of DNA nanoballs (DNB) were performed as previously described (Wu J. et al., 2025). The DNA was fragmented and tagged with adapter sequences using a DNA Library Prep Set (MGI Tech Co., Ltd., China). The library was then amplified by PCR, followed by cleanup and size selection with a DNA Clean Beads Kit (MGI Tech Co., Ltd., China) to eliminate very short library fragments. The MGIEasy circularization module V2.0 (MGI Tech Co., Ltd., China) was subsequently used to denature the double-stranded library into circular single-stranded DNA and create DNB. The DNB was loaded onto an MGISEQ-2000RS Sequencing Flow Cell and sequenced on the MGISEQ-2000 platform (MGI Tech Co., Ltd., China) using a paired-end (PE100) strategy with the MGISEQ-2000RS high-throughput sequencing kit. The raw FASTQ sequences were evaluated for quality using FastQC v0.11.7 (https://www.bioinformatics.babraham.ac.uk/projects/fastqc/). Quality control of the FASTQ reads involved filtering out low-quality reads and retaining high-quality “clean reads” for genome assembly. The filtration criteria included the removal of reads with low average base quality or a high proportion of N bases. *De novo* assembly was performed using a De Bruijn graph algorithm. The quality of the assembled sequences was assessed for N50, GC content, assembly size, sequencing depth, and contig counts using Quast v4.6.3 (https://github.com/ablab/quast/) (Supplementary Data S2).

### 2.4 Bioinformatic analysis

#### 2.4.1 Multilocus sequence typing (MLST) analysis

MLST analysis was conducted using PubMLST (https://pubmlst.org/). Of the 284 mPCR-confirmed *Salmonella* 1,4,[5],12:i:- isolates, 281 were assigned to ST34 and were included in the subsequent genomic analyses. The distribution of the 281 ST34 isolates by year was as follows: 19 isolates (6.8%) in 2019, 26 (9.3%) in 2020, 57 (20.3%) in 2021, 69 (24.6%) in 2022, and 110 (39.1%) in 2023.

#### 2.4.2 Analysis of ARGs and plasmids

Using the assembled genome sequences, ARGs were screened using the Resistance Gene Identifier (RGI 6.0.5) against the Comprehensive Antibiotic Resistance Database (CARD 4.0.1; https://card.mcmaster.ca/analyze/rgi). Both the minimum percentage identity and the query coverage thresholds were set to >80%. This analysis aimed to determine the prevalence of ARGs and point mutations in *Salmonella* 1,4,[5],12:i:- ST34 isolates from Guizhou Province. Furthermore, we compared the genotypic results with the AMR phenotypes to assess the agreement between genotype and phenotype in these isolates.

Plasmid replicons were identified using PlasmidFinder v2.1 (https://cge.food.dtu.dk/services/PlasmidFinder/), with parameters set to a minimum identity threshold of >95% and a minimum coverage threshold of >60%. These criteria were applied to determine the current prevalence of plasmids among *Salmonella* 1,4,[5],12:i:- ST34 in Guizhou Province. Furthermore, PlasmidHunter v1.3 (https://github.com/tianrenmaogithub/PlasmidHunter) (Tian et al., 2024) was used to predict plasmid contigs with default parameters. An ARG was classified as plasmid-associated if it was located on a contig predicted as plasmid-derived by PlasmidHunter v1.3. Finally, a correlation analysis was conducted to explore the associations between plasmid-associated ARGs and plasmid replicon types.

#### 2.4.3 Detection of additional mobile genetic elements (MGEs)

To investigate additional MGEs potentially involved in ARG dissemination, all 281 genome assemblies were screened for integron integrases (IntI1, IntI2, IntI3) and two insertion sequence-associated transposases (ISEcp1, IS*26*). Genes were predicted for each assembly using Pyrodigal v3.7.1 (https://github.com/althonos/pyrodigal) in single-genome mode with self-training on each assembly. The predicted proteins were then compared against curated reference sequences from UniProtKB (https://www.uniprot.org/) for class 1, 2, and 3 integron integrases, ISEcp1/IS*1380*-family transposase, and IS*26*/IS*6*-family transposase, by global pairwise alignment using edlib v1.3.9 (https://github.com/martinsos/edlib). Candidate proteins were restricted to those within ± 30% of the reference protein length. A locus was considered present when the best-matching predicted protein showed ≥70% identity over ≥70% of the reference length. Scores below this threshold were interpreted as absence or as divergence beyond confident detection.

#### 2.4.4 Targeted analysis of virulence-associated genes

A targeted panel of 34 canonical *Salmonella* virulence-associated loci was screened using the same gene-calling and alignment approach described in Section 2.4.3 against the *Salmonella* Typhimurium LT2 reference proteome (UniProtKB, proteome ID: UP000001014). The panel spanned the SPI-1 and SPI-2 type III secretion systems and associated effectors/regulators (*invA, invG, invF, prgH, prgK, sipB, sipC, hilA, hilD, sprB, sopB, sopE2, avrA, ssaV, ssaU, sseB, ssrA, ssrB, spiC, sifA*), fimbrial and biofilm adhesins (*fimA, fimH, lpfA, csgA, csgD*), the PhoP/PhoQ two-component regulatory system (*phoP, phoQ*), the stationary-phase sigma factor RpoS (*rpoS*), magnesium transport (*mgtB, mgtC*), iron acquisition (*entB, fepA*), the plasmid-borne virulence gene (*spvC*), and one Gifsy-2 prophage-linked locus (*sodCI*).

To assess whether ARGs and virulence determinants co-localize on the same plasmid, the contig harboring each of the 34 virulence-associated loci was identified and cross-referenced against the isolate's set of plasmid-associated contigs predicted by PlasmidHunter v1.3. A virulence-associated locus and an ARG were considered co-localized if they resided on the same plasmid contig.

### 2.5 Molecular epidemiological analysis

Information on the *Salmonella* ST34 isolates was exported from EnteroBase (https://enterobase.warwick.ac.uk/). First, an initial screening of records of *Salmonella* Typhimurium and its monophasic variants was conducted using Microsoft Excel 2021. We then excluded isolates with incomplete information on year, country, or source, and those that did not have a corresponding Sample ID in NCBI. Given computational limitations, we stratified the remaining isolates by country and selected representative isolates with available genome sequences in NCBI (https://www.ncbi.nlm.nih.gov/), yielding a final set of 465 isolates.

Among these 465 isolates, 90 were identified as *Salmonella* Typhimurium, whereas 375 were *Salmonella* 1,4,[5],12:i:-. The isolation years spanned from 1996 to 2023, and the isolates originated from 17 countries/territories across Asia, North America, Europe, and Oceania. The sources of the isolates included humans, food (staple foods and seafood), livestock (pork and beef), poultry (chicken), animals (including aquatic, wild, and companion animals), and environmental samples (Supplementary Data S3). CgMLST and cgSNP analyses were performed for these 465 isolates and the 281 *Salmonella* 1,4,[5],12:i:- isolates from Guizhou Province.

#### 2.5.1 Core genome multilocus sequence typing (cgMLST) analysis

CgMLST analysis was performed using cgMLSTFinder v1.2.0 (https://cge.food.dtu.dk/services/cgMLSTFinder/) with default parameters against the built-in *Salmonella* cgMLST scheme (3,002 loci), and cgSTs were conducted on the genome sequences of 281 *Salmonella* 1,4,[5],12:i:- ST34 isolates from Guizhou Province. A neighbor-joining (NJ) tree was constructed based on the cgMLST alleles of 281 *Salmonella* 1,4,[5],12:i:- ST34 isolates using the pairwise allele difference matrix. The tree was then visualized and annotated using iTOL v7 (https://itol.embl.de/) to reveal the genetic diversity of *Salmonella* 1,4,[5],12:i:- ST34 in Guizhou Province.

CgMLSTFinder v1.2.0 was also used to analyze 746 ST34 *Salmonella* genome sequences, including the 281 Guizhou isolates and 465 sequences downloaded from NCBI. This analysis generated a 3,002-locus allele profile for each genome (Supplementary Data S4). To investigate the origins of *Salmonella* 1,4,[5],12:i:- ST34 in Guizhou Province, a minimum spanning tree (MST) was constructed using GrapeTree v1.5.0 with the MSTreeV2 algorithm under default clustering settings, ignoring missing values.

#### 2.5.2 Core genome single-nucleotide polymorphism (cgSNP) analysis

The cgSNP analysis was performed using the BAIYI MicroGeno Platform (v5.4; Hangzhou Baiyi Technology Co., Ltd., http://www.baiyi-tech.cn/). A cgSNP analysis of 746 ST34 *Salmonella* genome sequences was conducted with snippy v4.6.0 using default settings (https://github.com/tseemann/snippy) to obtain the genetic distances, and the *Salmonella* Typhimurium LT2 genome (GenBank accession number: NC_003197) was used as a reference. Core SNPs were identified using the snippy-core function. A maximum-likelihood (ML) tree was constructed with IQ-TREE v2.4.0 (http://www.iqtree.org) using default model selection (-m MFP) and 1,000 ultrafast bootstrap replicates. To visualize the evolutionary relationships of *Salmonella* 1,4,[5],12:i:- ST34 in Guizhou Province, GrapeTree v1.5.0 (https://github.com/achtman-lab/GrapeTree) and iTOL v7 (https://itol.embl.de/) were used.

### 2.6 Gene content overview

Protein-coding genes were predicted for all 281 assemblies using Pyrodigal v3.7.1 in single-genome mode. For each genome, the total number of predicted genes was recorded as a descriptive measure of coding capacity. In addition, the established 3,002-locus *Salmonella* enterica cgMLST scheme (EnteroBase) was used as the core-genome reference; the difference between the total predicted gene count and 3,002 was used as an approximation of accessory gene content.

### 2.7 Statistical analysis

Statistical analyses were performed using R version 4.3.2 (R Foundation for Statistical Computing, Vienna, Austria). The Cochran-Armitage trend test (from the CATT package) was used to analyze temporal changes in resistance rates to 17 antibiotics from 2019 to 2023. Agreement between ARGs and resistance phenotypes was assessed by calculating the consistency rate, Cohen's *Kappa* statistic, sensitivity, and specificity (using the epiR package). A *Kappa* value of ≥0.75 indicated excellent agreement, between 0.40 and 0.75 indicated good agreement, and ≤ 0.40 indicated poor agreement. The association between IncHI2/IncHI2A plasmid carriage and the presence of CIA-ARGs in Clades 4–5 was evaluated using Pearson's chi-squared test. Associations between plasmid-associated ARGs and plasmid replicons were evaluated using Fisher's exact test, with the strength of association measured by the phi coefficient. Differences in the prevalence of individual ARGs and plasmid replicons across the 10 predominant cgSTs were assessed using Fisher's exact test with Monte Carlo simulation (2,000 replicates). *P*-values were adjusted for multiple testing using the Benjamini-Hochberg method. Differences in ARG counts between IntI1-positive and IntI1-negative isolates were compared using the Mann-Whitney *U*-test. A *P*-value of < 0.05 was considered statistically significant.

## 3 Results

### 3.1 Antimicrobial resistance phenotype

**Figure 1:**
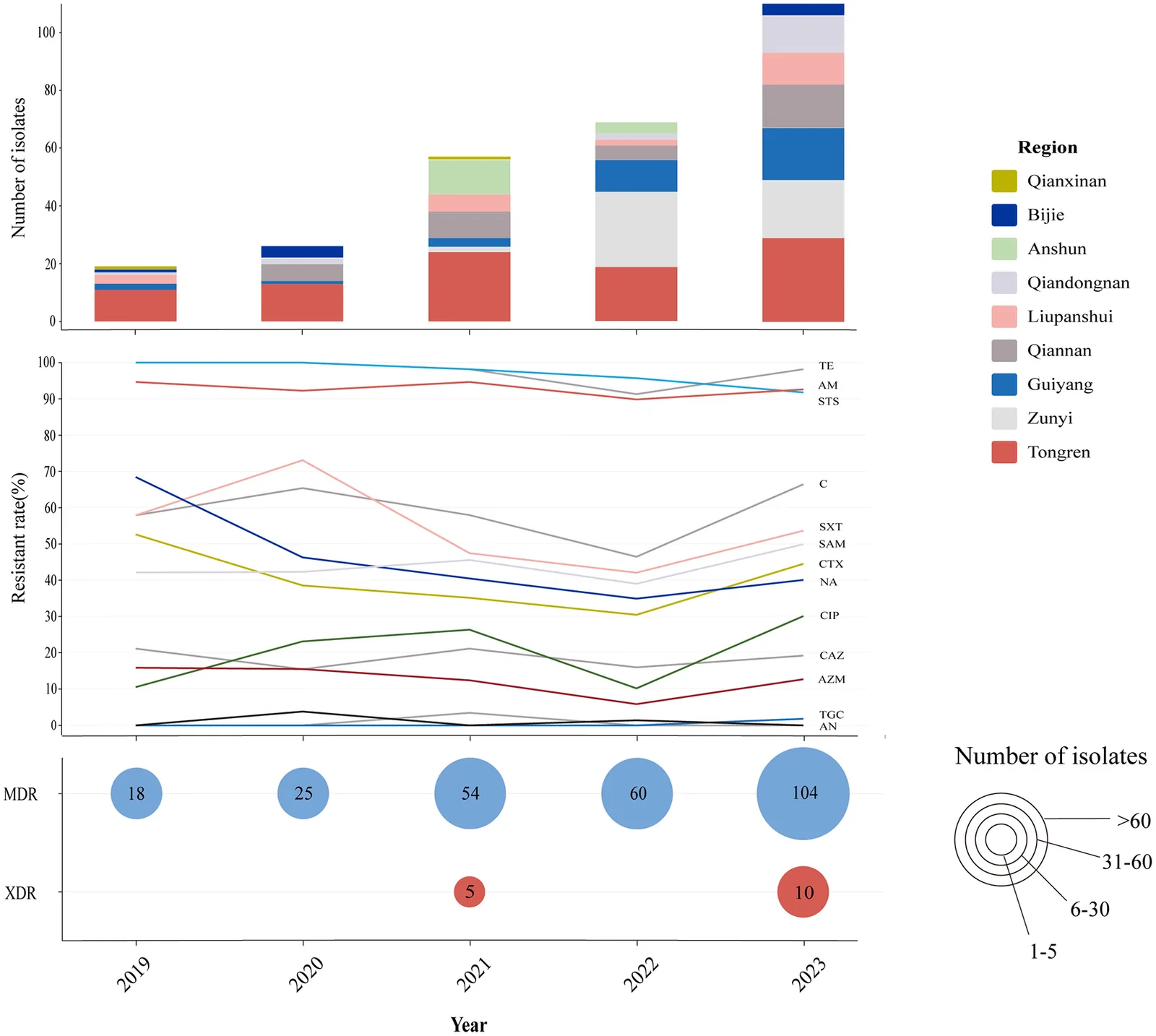
Regional distribution and resistance phenotype analysis of *Salmonella* 1,4,[5],12:i:- ST34 in Guizhou Province from 2019 to 2023. The upper panel shows the number of *Salmonella* 1,4,[5],12:i:- isolates collected in each year from nine cities (prefectures) in Guizhou Province; The middle panel displays the changes in resistance rates; the lower panel shows the numbers of MDR and XDR *Salmonella* 1,4,[5],12:i:-.

A total of 281 *Salmonella* 1,4,[5],12:i:- ST34 isolates were collected from six cities and three prefectures in Guizhou Province. Tongren contributed the greatest number of isolates (96/281), followed by Zunyi (48/281), Guiyang (35/281), and Qiannan (35/281). From 2019 to 2023, Zunyi had the highest number of isolates in 2022, while Tongren accounted for the highest proportion in all other years (Figure 1).

Antibiotic susceptibility testing revealed that all isolates were resistant to at least one antibiotic. The highest resistance rates were observed for tetracycline (96.8%, 272/281), streptomycin (95.4%, 268/281), and ampicillin (92.5%, 260/281). All isolates were susceptible to meropenem and ceftazidime/avibactam. Additionally, the resistant rates for ciprofloxacin, azithromycin, ceftazidime, and cefotaxime were 22.4% (63/281), 11.4% (32/281), 18.5% (52/281), and 39.1% (110/281), respectively. Two colistin-resistant isolates, one ertapenem-resistant isolate, and two tigecycline-resistant *Salmonella* 1,4,[5],12:i:- isolates were identified between 2021 and 2023 (Supplementary Data S5). Cochran-Armitage trend tests indicated no significant temporal trend in resistance rates from 2019 to 2023 (*P* > 0.05). However, a notable decrease in resistance to all antibiotics, except for amikacin, was observed in 2022, followed by an increase in most resistance rates in 2023.

Among the 281 *Salmonella* 1,4,[5],12:i:- isolates analyzed, 261 (92.9%) were classified as MDR. The number of MDR isolates has increased annually from 2019 to 2023. Additionally, 15 isolates (5.3%) were categorized as XDR and remained susceptible to only one or two antimicrobial categories. Among these XDR isolates, five were identified in 2021 and 10 in 2023. The distribution of XDR isolates was as follows: Zunyi (46.7%, 7/15), Qiannan (20.0%, 3/15), Guiyang (13.3%, 2/15), Tongren (13.3%, 2/15), and Liupanshui (6.7%, 1/15).

A total of 96 unique AMR patterns were identified from 281 *Salmonella* 1,4,[5],12:i:- isolates in Guizhou Province (Supplementary Data S5). The dominant AMR pattern was TE + STS + AM (14.9%, 42/281), followed by TE+STS (4.3%, 12/281) and C + SXT + TE + STS + AM (3.9%, 11/281). Although some regional variation was observed, the TE + STS + AM pattern was predominant in six of the nine cities (prefectures).

### 3.2 Distribution of ARGs and point mutations

**Figure 2:**
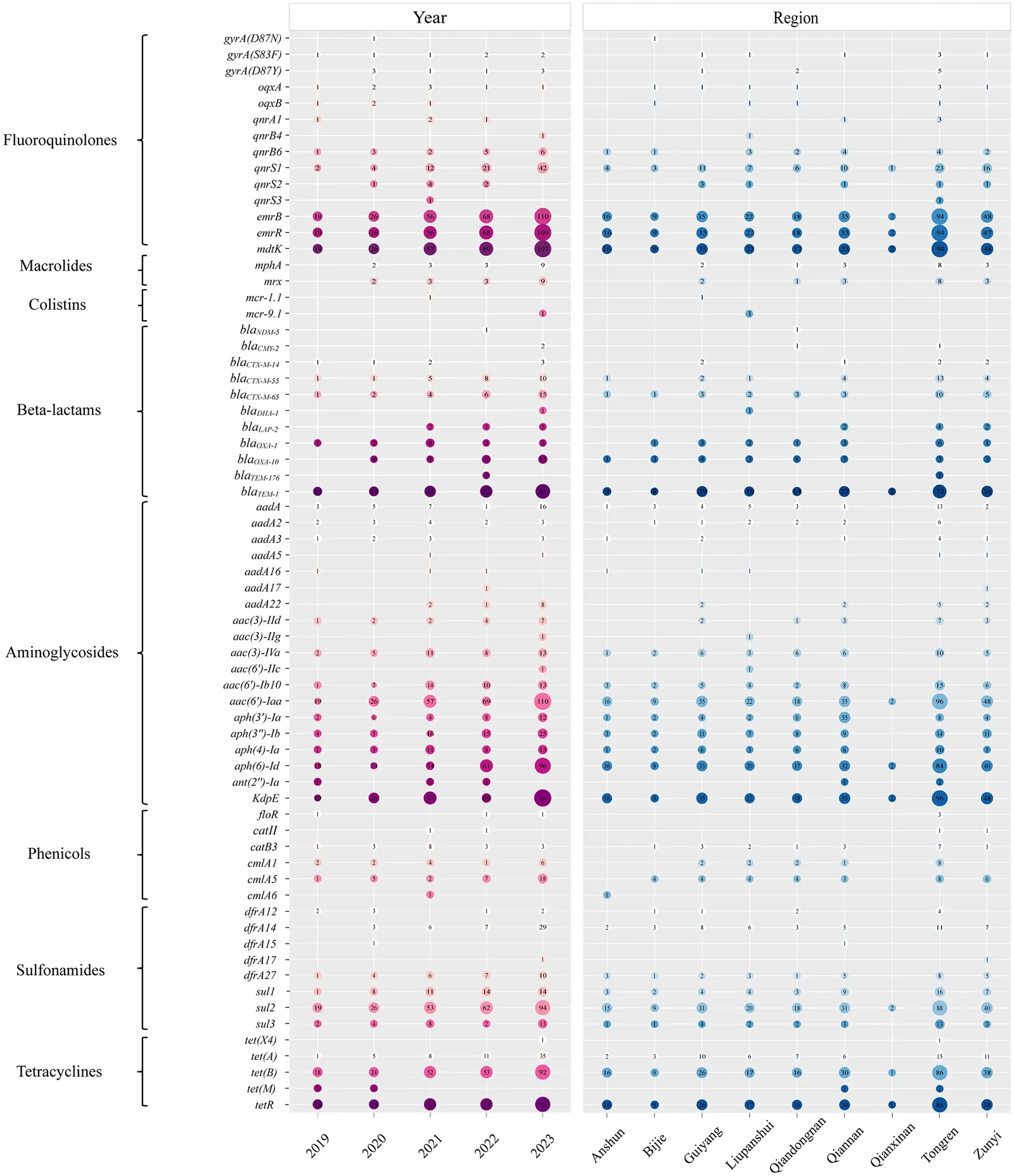
Distribution of ARGs and point mutations carried by *Salmonella* 1,4,[5],12:i:- ST34 in Guizhou Province. From left to right, this figure details the temporal and regional distributions of different ARGs and point mutations.

Between 2019 and 2023, the types and total numbers of ARGs associated with CIAs displayed a year-by-year increasing trend (Figure 2). Notably, the macrolide resistance genes *mphA* and *mrx* were first identified in 2020. The 3GC resistance gene *bla*_*TEM*−176_ and the carbapenem resistance gene *bla*_*NDM*−5_ were first detected in 2022, followed by the fluoroquinolone resistance gene *qnrB4* in 2023. The colistin resistance gene *mcr-1.1* was also detected in 2021, with *mcr-9.1* further identified in 2023.

The distribution of ARGs varied across the nine cities (prefectures) in Guizhou Province. The isolates from Tongren, Zunyi, Guiyang, and Qiannan exhibited greater diversity and quantity of ARGs. All of these isolates harbored fluoroquinolone resistance genes (*qnrS1, qnrS2*), a point mutation [*gyrA* (S83F)], macrolide resistance genes (*mphA, mrx*), and *ESBL* genes (*bla*_*CTX*−*M*−14_, *bla*_*CTX*−*M*−55_, *bla*_*CTX*−*M*−65_). Furthermore, the carbapenem resistance gene *bla*_*NDM*−5_ was exclusively detected in Qiandongnan, whereas the colistin resistance genes *mcr-1.1* and *mcr-9.1* were identified in Guiyang and Liupanshui, respectively.

Similarly, the types and total number of ARGs associated with traditional antibiotics increased from 2019 to 2023. Among these ARGs, aminoglycoside resistance genes (*aadA, aadA22*), phenicol resistance genes (*cmlA1, cmlA5*), sulfonamide resistance genes (*dfrA14, sul3*), and the tetracycline resistance gene *tet(A)* were detected more frequently in 2023. The tigecycline resistance gene *tet(X4)* was first detected in *Salmonella* 1,4,[5],12:i:- in Guizhou Province. Among the nine cities (prefectures), isolates from Tongren and Zunyi presented the highest diversity and abundance of ARGs, with *tet(X4)* being uniquely identified in Tongren.

### 3.3 Consistency analysis of ARGs and resistance phenotypes

**Figure 3:**
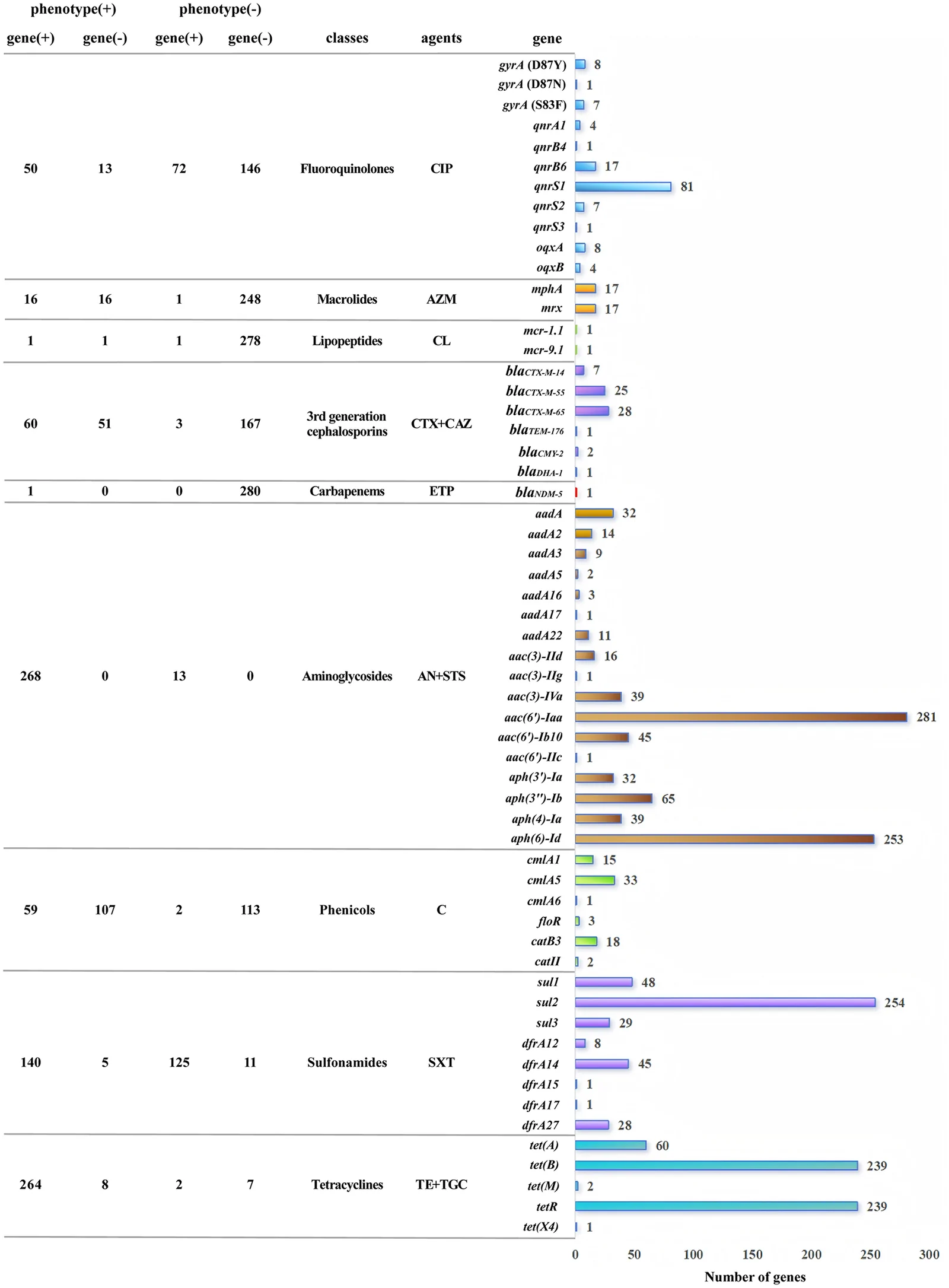
Consistency analysis of ARGs and resistance phenotypes in *Salmonella* 1,4,[5],12:i:- ST34 from Guizhou Province. For each category of antimicrobial, the phenotypic status was defined as positive [phenotype (+)] if at least one antibiotic within that category showed resistance. Similarly, the ARG status of each category was marked as positive [gene (+)] if any corresponding ARG was detected within that category.

The results of the analysis on ARGs and resistance phenotypes demonstrated high consistency for macrolides, 3GCs, carbapenems, and lipopeptides. The consistency rates for these antibiotics were as follows: 94.0% (264/281) for macrolides, 80.8% (227/281) for 3GCs, 100.0% (281/281) for carbapenems, and 99.3% (279/281) for lipopeptides. The *Kappa* statistics for these analyses were 0.62, 0.57, 1.00, and 0.50, respectively (Figure 3). For fluoroquinolones, the combined set of eight ARGs and three point mutations predicted ciprofloxacin resistance with a sensitivity of 79.4% (50/63). The overall consistency rate was 69.8% (196/281). Notably, one isolate carrying the *bla*_*NDM*−5_ showed complete agreement with the ertapenem-resistant phenotype (*Kappa* = 1.00). Another isolate carrying *mcr-1.1* exhibited strong agreement with the colistin resistance phenotype, with a consistency rate of 99.6% and a *Kappa* value of 0.67. In addition, the *mphA* and *mrx* genes were in good agreement with the azithromycin resistance phenotype (consistency rate = 94.0%, *Kappa* = 0.62), whereas *bla*_*CTX*−*M*−55_ showed 89.7% agreement with ceftazidime resistance (*Kappa* = 0.57).

For traditional antibiotics, including aminoglycosides, phenicols, sulfonamides, and tetracyclines, the consistency rates between resistance genotypes and phenotypes were 95.4% (268/281), 61.2% (172/281), 53.7% (151/281), and 96.4% (271/281), respectively. Among these antibiotics, tetracyclines showed good agreement (*Kappa* = 0.57). Aminoglycosides exhibited high sensitivity (100.0%, 268/268), and sulfonamides displayed a sensitivity of 96.6% (140/145). Phenicol demonstrated high specificity (98.3%, 113/115).

### 3.4 Prevalence of plasmids

The plasmid carriage rate among 281 *Salmonella* 1,4,[5],12:i:- ST34 isolates from Guizhou Province was 96.8% (272/281), with 26 plasmid replicon types identified. The most common plasmid replicon was IncQ1 (84.3%, 237/281), followed by IncHI2 and IncHI2A (26.3%, 74/281). Notably, all 74 isolates that carried IncHI2 also harbored IncHI2A. Additionally, a year-by-year increase in plasmid replicons was found in *Salmonella* 1,4,[5],12:i:- ST34 isolates in Guizhou Province, rising from 7 types in 2019 to 21 types in 2023.

**Figure 4:**
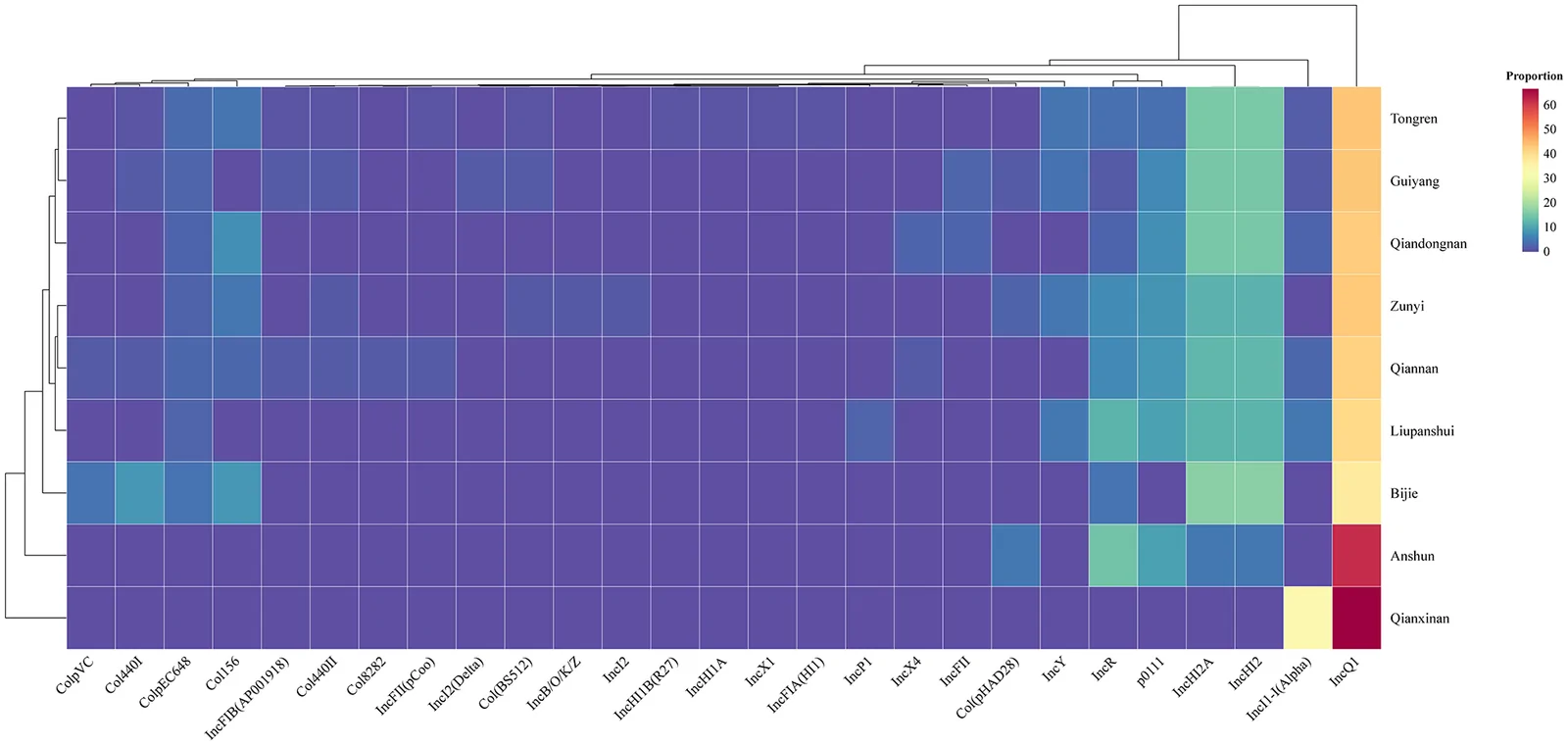
Heatmap of the regional distribution of plasmid replicons carried by *Salmonella* 1,4,[5],12:i:- ST34 in Guizhou Province. The color intensity represents the proportion of each plasmid replicon within each city (prefecture) in Guizhou Province.

The distribution of plasmids across cities (prefectures) in Guizhou Province is shown in Figure 4. IncQ1 was the dominant plasmid replicon in nine cities (prefectures). The second most common plasmid replicon was p0111 in Anshun and IncI1-I (Alpha) in Qianxinan, whereas IncHI2/IncHI2A ranked as the second most prevalent plasmid in the other cities. In addition, isolates from Tongren presented the greatest variety of plasmid replicons (17 types), followed by Guiyang and Qiannan, each with 14 types.

Statistical analysis of the combinations of plasmid incompatibility groups in the *Salmonella* 1,4,[5],12:i:- isolates from Guizhou Province revealed 49 combinations. Among these, 89 isolates (31.7%) carried only IncQ1, while 40 isolates (14.2%) carried the combination of IncQ1 and IncHI2/IncHI2A. The other combinations were relatively infrequent (< 10.0%). Notably, one isolate from Tongren (SM2023076), which carried a combination of IncQ1, Col156, IncFIA(HI1), IncHI1A, and IncHI1B(R27) plasmid incompatibility groups, also harbored the *tet(X4)* resistance gene (Supplementary Data S6).

### 3.5 Prediction of ARGs and point mutations on plasmids and chromosomes

**Figure 5:**
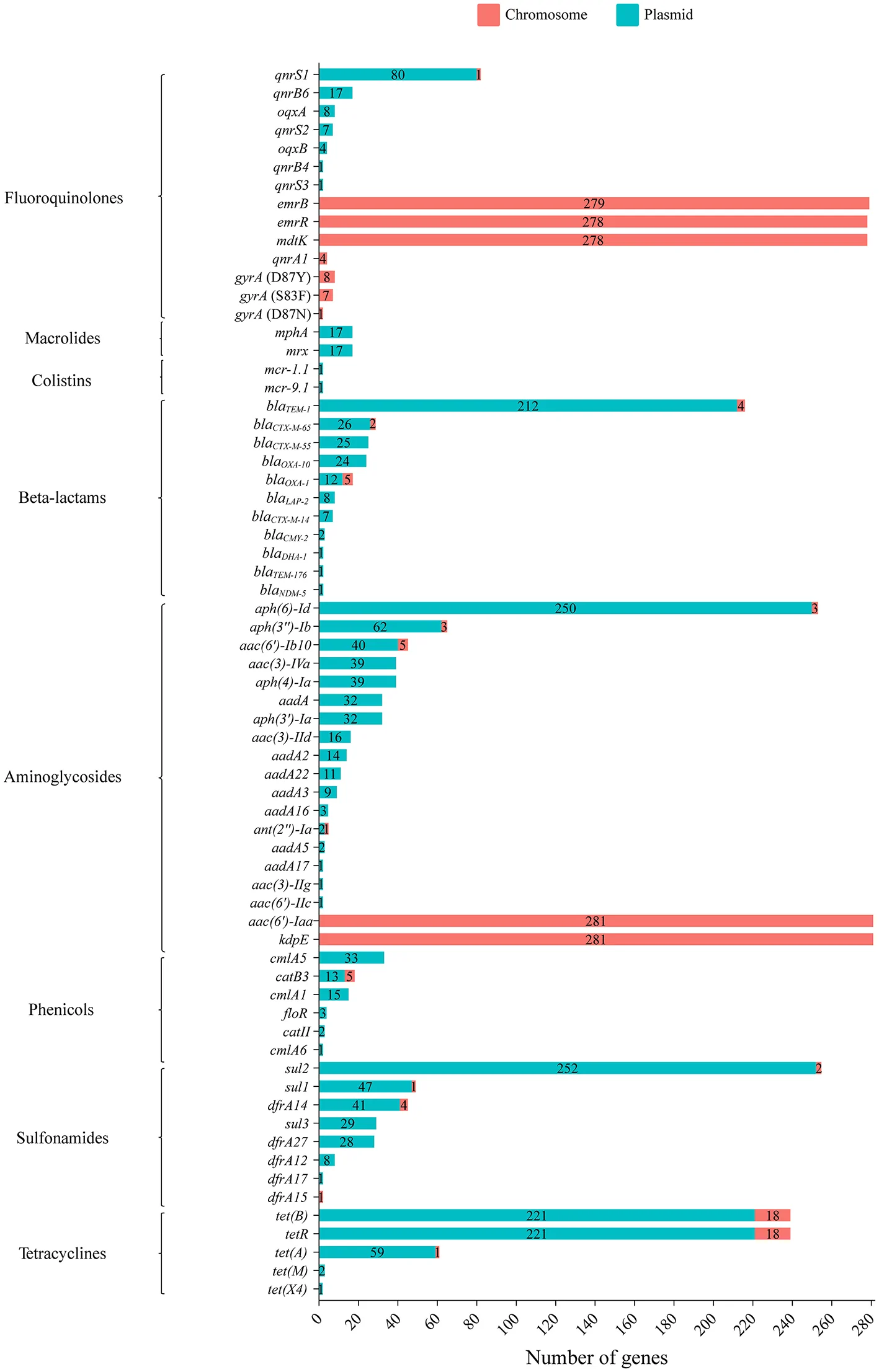
Prediction of ARGs and point mutations on plasmids and chromosomes of 281 *Salmonella* 1,4,[5],12:i:- ST34 isolates in Guizhou Province.

A total of 57 types of ARGs were predicted to be located on plasmid-associated contigs among *Salmonella* 1,4,[5],12:i:- ST34 isolates from Guizhou Province. The most prevalent ARGs in their respective antibiotic categories included the fluoroquinolone resistance gene *qnrS1* (28.5%, 80/281), the β-lactam resistance gene *bla*_*TEM*−1_ (75.4%, 212/281), the aminoglycoside resistance gene *aph(6)-Id* (89.0%, 250/281), the phenicol resistance gene *cmlA5* (11.7%, 33/281), the sulfonamide resistance gene *sul2* (89.7%, 252/281), and the tetracycline resistance gene *tet(B)* (78.6%, 221/281) (Figure 5). Notably, 96.7% (58/60) of the *ESBL* genes, *bla*_*CTX*−*M*_, were predicted to be located on plasmid-associated contigs. Additionally, fluoroquinolone resistance genes (*qnrS2, qnrS3, qnrB4, qnrB6, oqxA*, and *oqxB*), macrolide resistance genes (*mphA* and *mrx*), the tigecycline resistance gene [*tet(X4)*], the carbapenem resistance gene (*bla*_*NDM*−5_), and colistin resistance genes (*mcr-1.1* and *mcr-9.1*) were all predicted to be plasmid-associated.

In contrast, 22 types of ARGs and three distinct point mutations were predicted to be located on chromosome-associated contigs. Among these ARGs, fluoroquinolone resistance genes (*qnrA1, emrB, emrR*, and *mdtK*), aminoglycoside resistance genes (*aac6*′*)-Iaa* and *kdpE*), and a sulfonamide resistance gene (*dfrA15*) were all predicted to be chromosome-associated. For the point mutations, all fluoroquinolone resistance mutations in *gyrA*, including *gyrA* (D87Y), *gyrA* (S83F), and *gyrA* (D87N), were predicted to be chromosome-associated.

### 3.6 Analysis of associations between plasmid-associated ARGs and plasmid replicons

**Figure 6:**
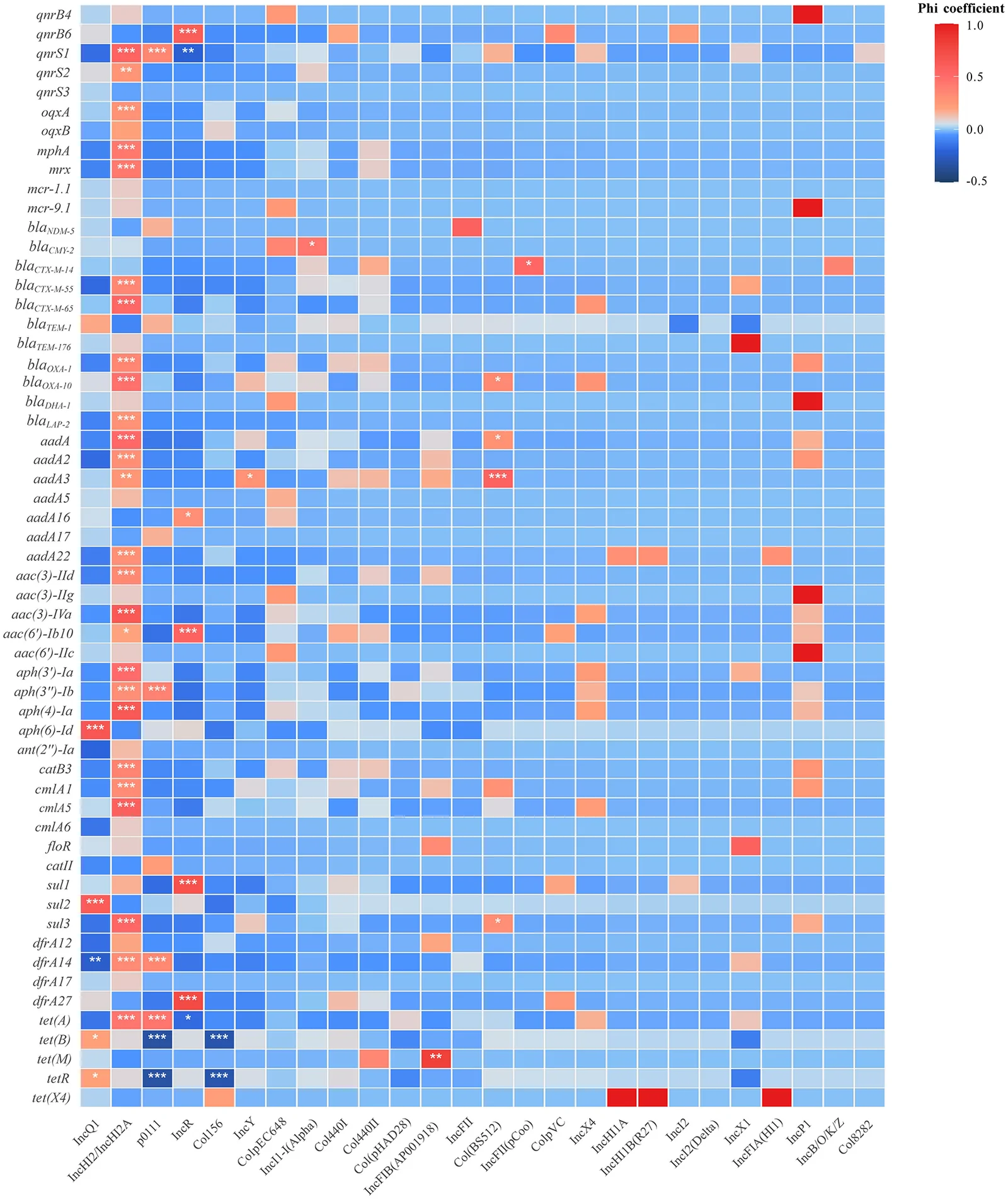
Analysis of associations between plasmid-associated ARGs and plasmid replicons. The scale represents phi coefficients ranging from −1 to 1; *P < 0.05; **P < 0.01; *** *P* < 0.001.

Further analyses of associations between plasmid-associated ARGs and various plasmid replicons were performed using Fisher's exact test (Figure 6). Associations were identified between several ARGs conferring resistance to CIAs and specific plasmid types. For example, the fluoroquinolone resistance gene *qnrB6* was strongly associated with the IncR plasmid (Φ = 0.60, *P* < 0.001). Among the 3GC-resistance genes, *bla*_*CMY*−2_ was associated with IncI1-I (Alpha) (Φ = 0.44, *P* < 0.05), and *bla*_*CTX*−*M*−14_ was associated with IncFII(pCoo) (Φ = 0.53, *P* < 0.05). Notably, multiple ARGs were associated with IncHI2/IncHI2A plasmids, including the fluoroquinolone resistance genes *qnrS1* (Φ = 0.55, *P* < 0.001), *qnrS2* (Φ = 0.27, *P* < 0.01), and *oqxA* (Φ = 0.29, *P* < 0.001); the macrolide resistance genes *mphA* and *mrx* (Φ = 0.42, *P* < 0.001); and the 3GC resistance genes *bla*_*CTX*−*M*−55_ (Φ = 0.35, *P* < 0.001) and *bla*_*CTX*−*M*−65_ (Φ = 0.53, *P* < 0.001).

Multiple ARGs conferring resistance to traditional antibiotics were also associated with IncHI2/IncHI2A plasmids. These genes included aminoglycoside resistance genes *aadA* (Φ = 0.52, *P* < 0.001) and *aac(3)-IVa* (Φ = 0.65, *P* < 0.001), the phenicol resistance gene *cmlA5* (Φ = 0.59, *P* < 0.001), the sulfonamide resistance gene *sul3* (Φ = 0.54, *P* < 0.001), and the tetracycline resistance gene *tet(A)* (Φ = 0.45, *P* < 0.001). Moreover, other associations were observed between specific ARGs and plasmids: *aadA3* was associated with Col(BS512) (Φ = 0.57, *P* < 0.001), *aac6*′*)-Ib10* with IncR *(*Φ = 0.56, *P* < 0.001), *aph(6)-Id* with IncQ1 (Φ = 0.66, *P* < 0.001), *sul1* with IncR (Φ = 0.69, *P* < 0.001), *sul2* with IncQ1 (Φ = 0.63, *P* < 0.001), *dfrA27* with IncR (Φ = 0.75, *P* < 0.001), and *tet(M)* with IncFIB(AP001918) (Φ = 0.82, *P* < 0.01). Additionally, a perfect positive association (Φ = 1.00) was observed between the *tet(X4)* gene and the replicons IncHI1A, IncHI1B(R27), and IncFIA(HI1).

### 3.7 Core genome multilocus sequence typing (cgMLST) analysis

#### 3.7.1 Prevalence of cgSTs and association with ARG and plasmid carriage

A total of 281 *Salmonella* 1,4,[5],12:i:- ST34 isolates from Guizhou were divided into 37 cgSTs (Supplementary Data S6). Among these, cgST52428 was the most common (32.7%, 92/281), followed by cgST137432 (13.9%, 39/281). The diversity of cgSTs increased annually, rising from seven in 2019 to 25 in 2023. CgST52428 consistently remained dominant.

**Figure 7:**
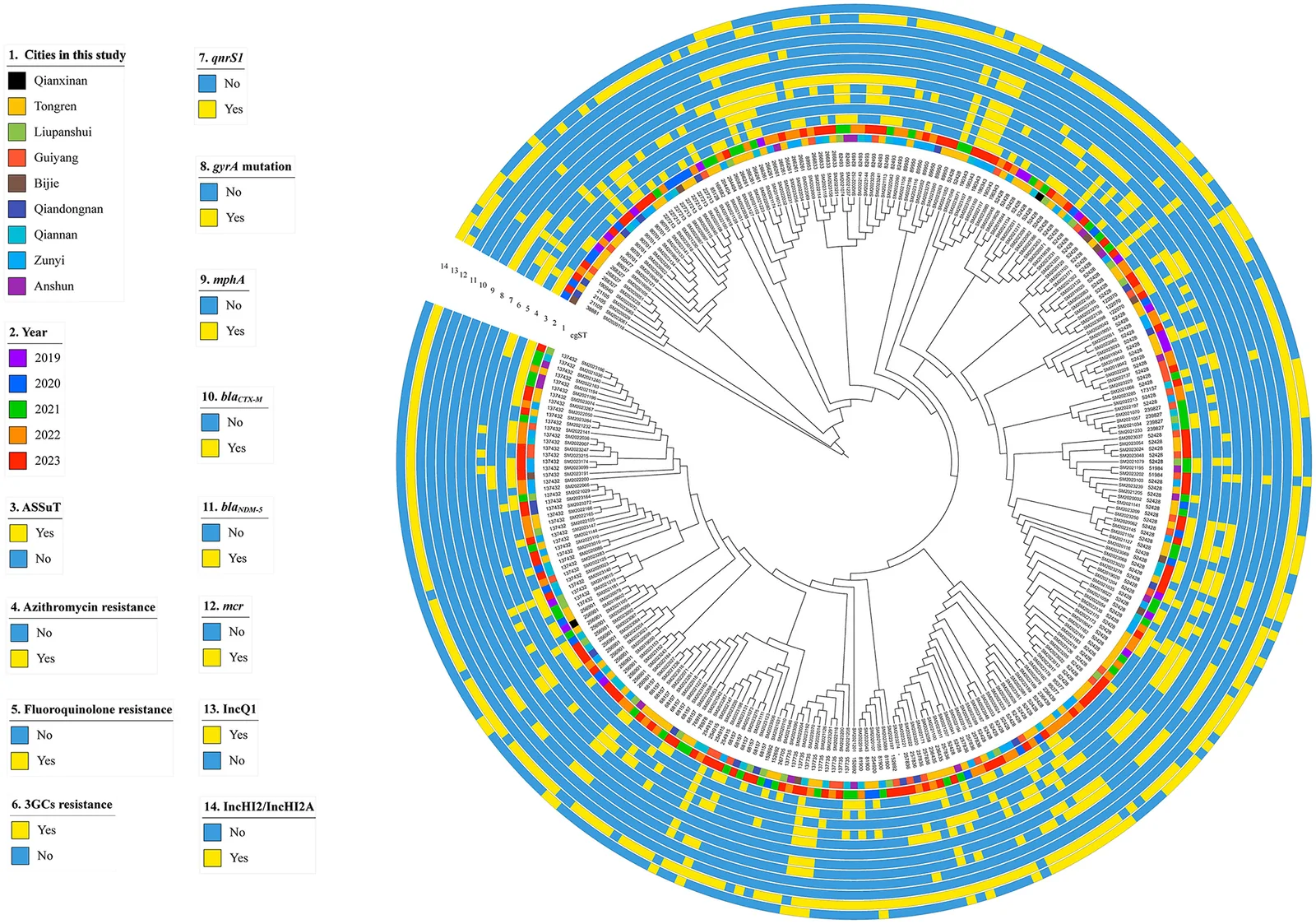
An NJ tree based on cgMLST alleles of 281 *Salmonella* 1,4,[5],12:i:-ST34 isolates from Guizhou Province. The innermost circle represents the cgST of these 281 sequences. Different colors along the concentric circles represent different epidemiological features of the isolates. From the inner circle to the outer circle, the features represented are as follows: 1. Location, 2. Year of isolation, 3. ASSuT resistance phenotype, 4. Azithromycin resistance, 5. Fluoroquinolone resistance, 6. 3GC resistance, 7. *qnrS1* gene presence, 8. *gyrA* mutation, 9. *mphA* gene presence, 10. *bla*_*CTX*−*M*_ gene presence, 11. *bla*_*NDM*−5_ gene presence, 12. *mcr* gene presence, 13. IncQ1 plasmid replicon, and 14. IncHI2/IncHI2A plasmid replicons. The resistance phenotypes are represented by circles 3–6; circles 7–12 represent ARGs, and circles 13–14 denote plasmid replicons.

An NJ tree was constructed based on the cgMLST allele profile of 281 *Salmonella* 1,4,[5],12:i:-ST34 isolates from Guizhou Province (Figure 7). CgST52428 was the most common among ASSuT, fluoroquinolone, azithromycin, and 3GC-resistant isolates, accounting for 31.1% (42/135), 34.9% (22/63), 37.5% (12/32), and 38.7% (43/111), respectively. CgST137432 was the second most common type for ASSuT (17.8%, 24/135) and fluoroquinolone resistance (12.7%, 8/63); cgST137735 (12.5%, 4/32) and cgST266261 (8.1%, 9/111) ranked second among azithromycin- and 3GC-resistant isolates, respectively. Analysis of the distribution of specific ARGs and plasmids showed that cgST52428 accounted for the largest proportion of isolates carrying *qnrS1* (38.3%, 31/81), *mphA* (47.1%, 8/17), *bla*_*CTX*−*M*_ (46.7%, 28/60), *gyrA* mutations (43.8%, 7/16), and the IncHI2/IncHI2A plasmid (41.3%, 38/92). The isolate harboring *bla*_*NDM*−5_ belonged to cgST257836, while the isolates carrying *mcr-1.1* and *mcr-9.1* were classified as cgST68157 and cgST52428, respectively.

**Figure 8:**
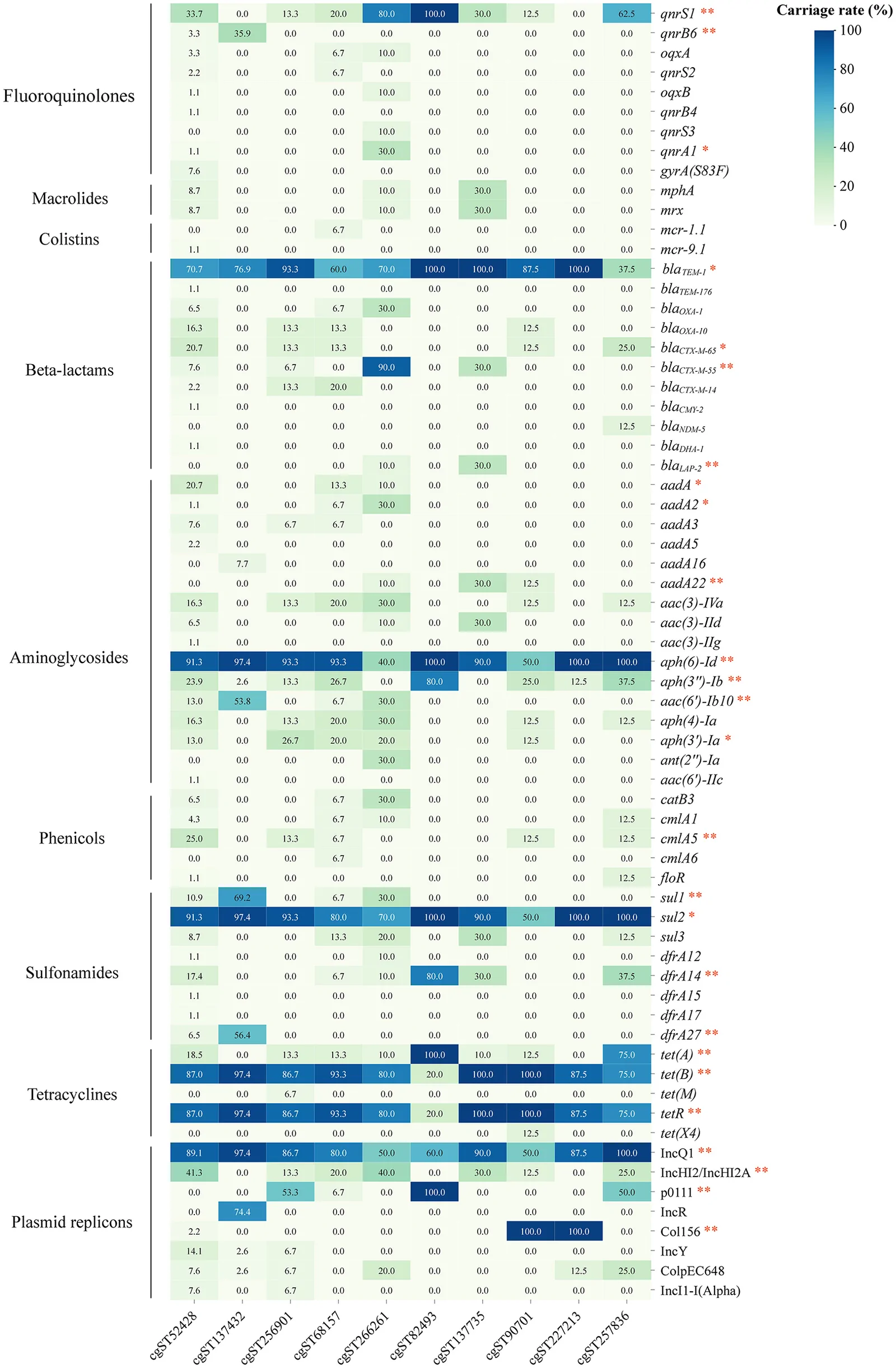
Prevalence of ARGs and plasmid replicons among the ten predominant cgSTs of *Salmonella* 1,4,[5],12:i:- ST34 in Guizhou Province. The heatmap shows the carriage rate (%) of each ARG and plasmid replicon within the corresponding cgST. Asterisks following their names denote statistically significant differences in prevalence across the ten cgSTs (Fisher's exact test with Benjamini-Hochberg correction; *P < 0.05; **P < 0.01).

To further characterize the ARG and plasmid carriage within each predominant cgST, we compared their prevalence across the 10 most common cgSTs, which together represented 76.5% (215/281) of all isolates. Significant heterogeneity in distribution was observed for most ARGs and plasmids (Figure 8). CgST52428 was characterized by the concurrent presence of *qnrS1* (33.7%, 31/92), *bla*_*CTX*−*M*−65_ (20.7%, 19/92), *cmlA5* (25.0%, 23/92), and the IncHI2/IncHI2A plasmid (41.3%, 38/92). CgST137432 displayed a distinct ARG profile, with high carriage of *qnrB6* (35.9%, 14/39), *aac(6*′*)-Ib10* (53.8%, 21/39), *sul1* (69.2%, 27/39), *dfrA27* (56.4%, 22/39), and the IncR plasmid (74.4%, 29/39). CgST266261, although comprising only 10 isolates, exhibited an extremely high prevalence of *bla*_*CTX*−*M*−55_ (90.0%) and multiple aminoglycoside ARGs (e.g., *aadA2, ant(2*″*)-Ia*, each 30.0%). CgST82493 showed distinct enrichment of *tet(A)* (100.0%, 10/10), *dfrA14* (80.0%, 8/10), and the p0111 plasmid (100.0%, 10/10).

#### 3.7.2 Minimum spanning tree (MST) analysis based on 3,002 allele profiles

**Figure 9:**
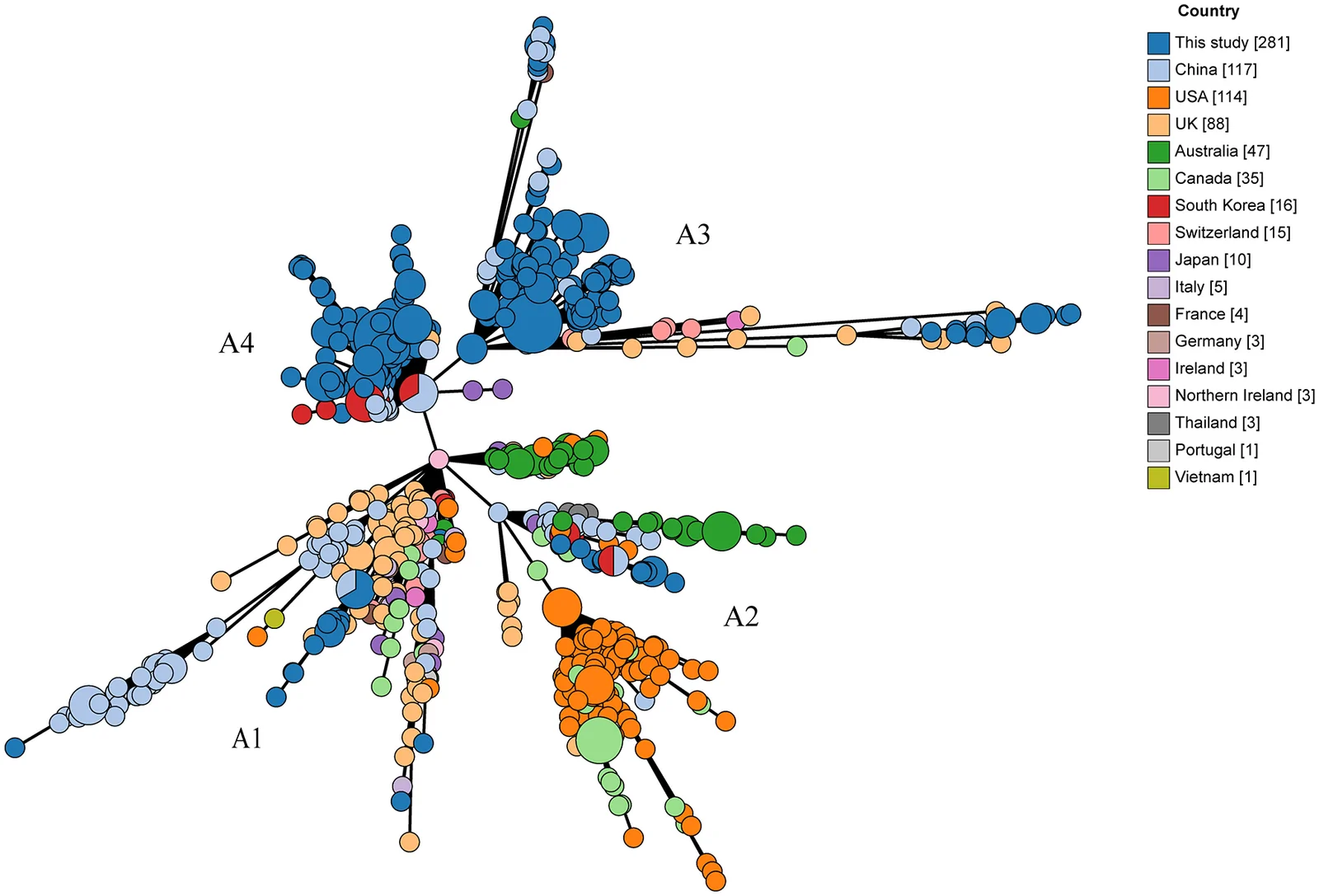
An MST of 746 ST34 *Salmonella* genome sequences (including 281 from Guizhou and 465 downloaded from NCBI) was constructed using the MSTreeV2 algorithm in GrapeTree based on 3,002 core-genome allele profiles. A total of 746 *Salmonella* ST34 isolates were grouped into four clusters (A1-A4). The MST incorporated genome sequences from 17 different countries/territories, with each country represented by a distinct color. The genome sequences of isolates from Guizhou were labeled in dark blue.

To explore the genetic relationships of *Salmonella* 1,4,[5],12:i:- ST34 in Guizhou Province, we performed an MST analysis using 746 *Salmonella* ST34 genome sequences-−465 from various sources and 281 specifically from Guizhou Province (Figure 9). The MST analysis resulted in the identification of four clusters (A1–A4). A majority (88.6%, 249/281) of the isolates from Guizhou were grouped into two clusters: A3 (43.4%, 122/281) and A4 (45.2%, 127/281). In cluster A3, many isolates from Guizhou exhibited short genetic distances to isolates from other parts of China, and some were similar to isolates from the United Kingdom. For instance, one isolate from Qiandongnan (SM2023065) was found to differ from a pork-derived isolate from Shanghai (SH12SF020) by five alleles. Another isolate from Tongren (SM2020108) differed from a United Kingdom isolate (918416) by 13 alleles. In cluster A4, the genetic relationships were similar, with Guizhou isolates showing short distances from isolates from other regions in China and South Korea. For example, one Tongren (SM2019051) isolate differed by one allele from a pork-derived isolate in Sichuan Province (SH16SF1005), and another Tongren (SM2020042) isolate differed by one allele from an isolate from South Korea (sporadic25). In addition, a few isolates from Guizhou were found in clusters A1 and A2. The isolates in cluster A1 showed close genetic relatedness with the isolates from Sichuan Province and the United Kingdom, while those in cluster A2 exhibited close relatedness with the isolates from Canada and South Korea.

### 3.8 Core genome single-nucleotide polymorphism (cgSNP) analysis

**Figure 10:**
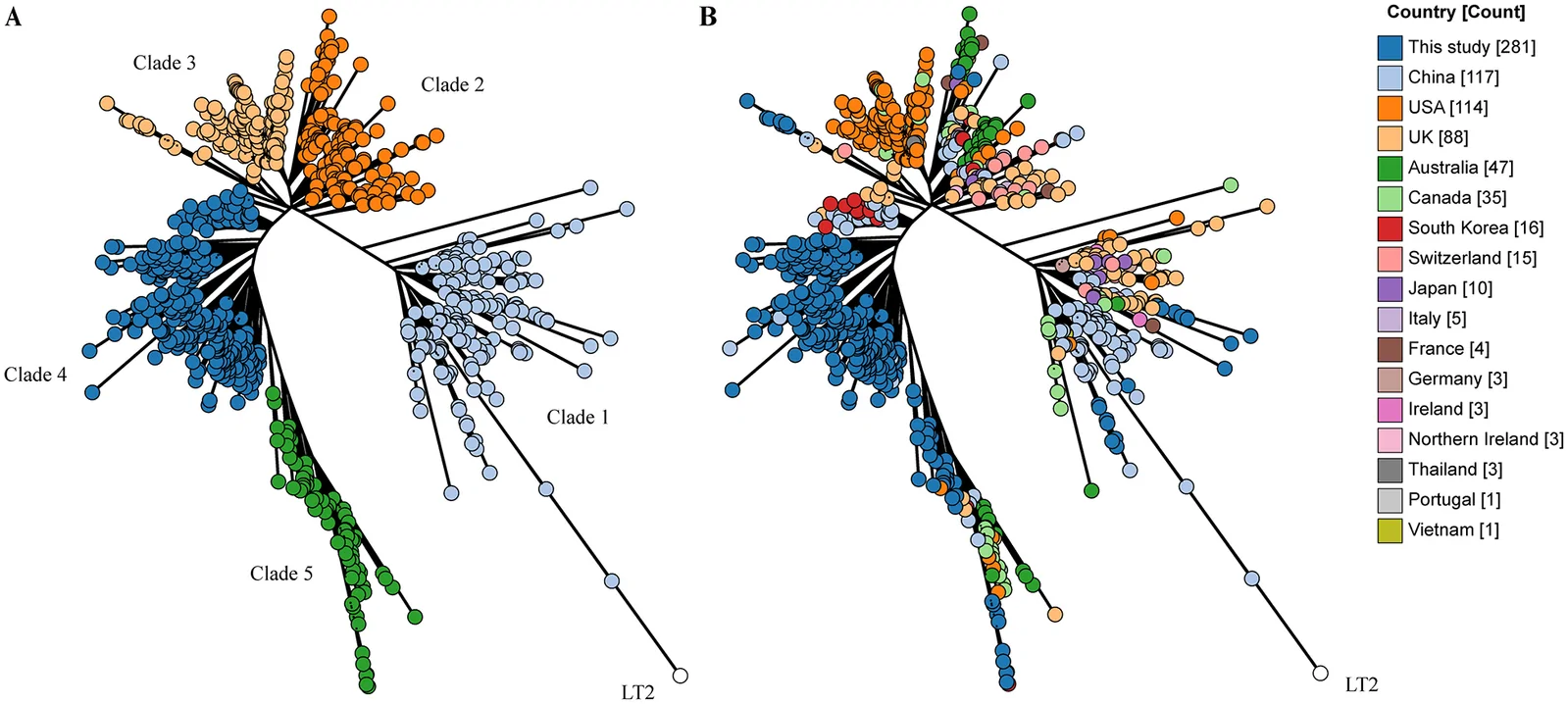
The ML tree of 746 ST34 *Salmonella* genome sequences (including 281 from Guizhou and 465 downloaded from NCBI) was constructed using cgSNP. **(A)** The sequence data were visualized using GrapeTree. The 746 *Salmonella* ST34 genome sequences were clustered into five distinct clades, represented by different colors. The reference strain LT2 was shown without color, and the dashed line indicated genetic distances exceeding 0.0025. **(B)** GrapeTree visualization was further colored by country. Sequences from Guizhou were highlighted in dark blue.

To further investigate the evolutionary relationships of *Salmonella* 1,4,[5],12:i:- ST34 isolates from Guizhou Province, we performed a comprehensive cgSNP analysis using 746 *Salmonella* ST34 genome sequences. The isolates were categorized into five distinct clades (Figure 10A), with the following distribution: Clade 1: 165 isolates (22.1%), Clade 2: 132 isolates (17.7%), Clade 3: 119 isolates (16.0%), Clade 4: 257 isolates (34.4%), and Clade 5: 73 isolates (9.8%). Analysis of the years when the isolates were collected indicated that Clade 1 contained the earliest isolates, dating back to 1996, while the other clades primarily consisted of isolates collected from 2005 onward. A majority of the isolates (90.6%, 299/330) in Clades 4 and 5 were gathered in 2019 or later (Supplementary Data S7). Further genetic distance analysis revealed that Clade 1 was phylogenetically closest to the reference strain LT2, while Clades 4 and 5 exhibited the most significant genetic distance from LT2 (Figure 10A). When the serotype distribution was examined, all 90 *Salmonella* Typhimurium sequences from NCBI were clustered within Clade 1, while Clades 2 to 5 were contained exclusively by *Salmonella* 1,4,[5],12:i:-.

Among the *Salmonella* 1,4,[5],12:i:- isolates from Guizhou, 77.2% (217/281) belonged to Clade 4, representing 84.4% (217/257) of all the isolates in that clade (Figure 10B). Clade 4 also included genetically similar isolates from other provinces. For example, one isolate from Tongren (SM2021042) differed by 184 SNPs from a Zhejiang isolate (SAL1706), and another from Qiannan (SM2021233) differed by 202 SNPs from a Fujian isolate (SH18G2954). Additionally, 12.8% (36/281) of the isolates from Guizhou clustered in Clade 5, which also included isolates from South Korea, Europe, and the USA. For instance, an isolate from Tongren (SM2023256) differed by 152 SNPs from a Guangdong isolate (SH18SF289), while another from Tongren (SM2021223) differed by 176 SNPs from a South Korean isolate (sporadic14).

**Figure 11:**
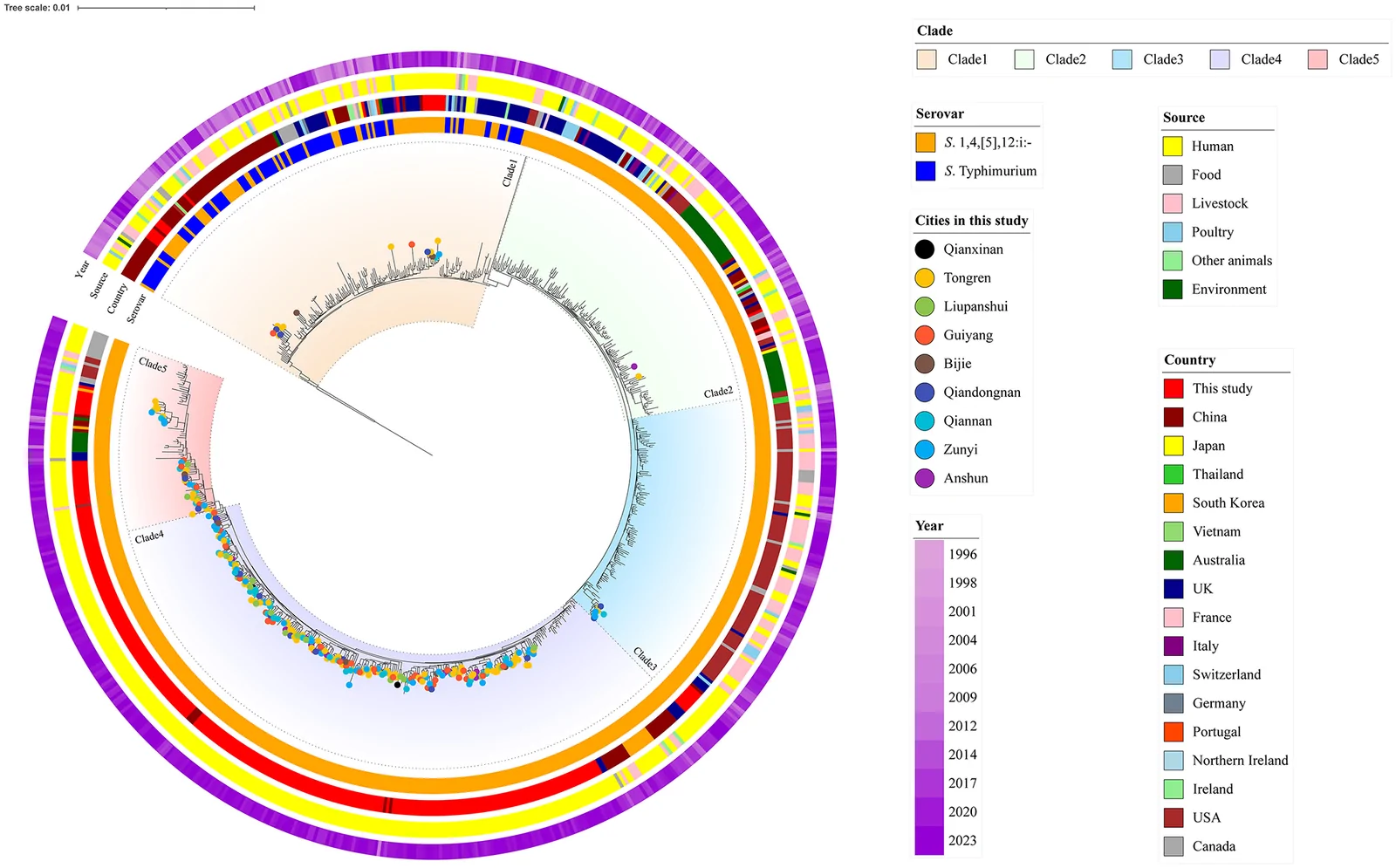
Population structure of the ST34 *Salmonella* ML phylogenetic tree based on 281 genome sequences from this study and 465 publicly available genome sequences from NCBI. Visualization via iTOL included a scale bar representing the number of substitutions per site per genome. The tree nodes were color-coded according to nine cities (prefectures) in Guizhou Province. Epidemiological information for the isolates was displayed in concentric circles, arranged from innermost to outermost: 1. Serovar, 2. Country, 3. Source of isolation, 4. Year of isolation.

Analysis of the source of the *Salmonella* ST34 isolates revealed that human-derived isolates from Guizhou predominantly clustered in Clade 4 with isolates from poultry, livestock, food, and other animals (Figure 11). One human-derived isolate from Tongren (SM2023256) differed by 152 SNPs from a pork-derived isolate from Guangdong (SH18SF289). In Clade 4, human-derived isolates from Guizhou were found alongside those from livestock and food, such as a human-derived isolate from Liupanshui (SM2023187) differing by 156 SNPs from a pork-derived isolate from Sichuan (SH16SF1005) and another from Zunyi (SM2022062) differing by 168 SNPs from a pork-derived isolate from Beijing (SH17SF512). The environment-derived isolates were distributed across Clades 1 to 3, with one Shanghai isolate (SH10SF279) differing by 224 SNPs from a human-derived isolate from Tongren (SM2020108). This clear structure emphasizes the evolutionary relationships, geographic distribution, and potential sources of *Salmonella* 1,4,[5],12:i:- ST34 within the dataset.

**Figure 12:**
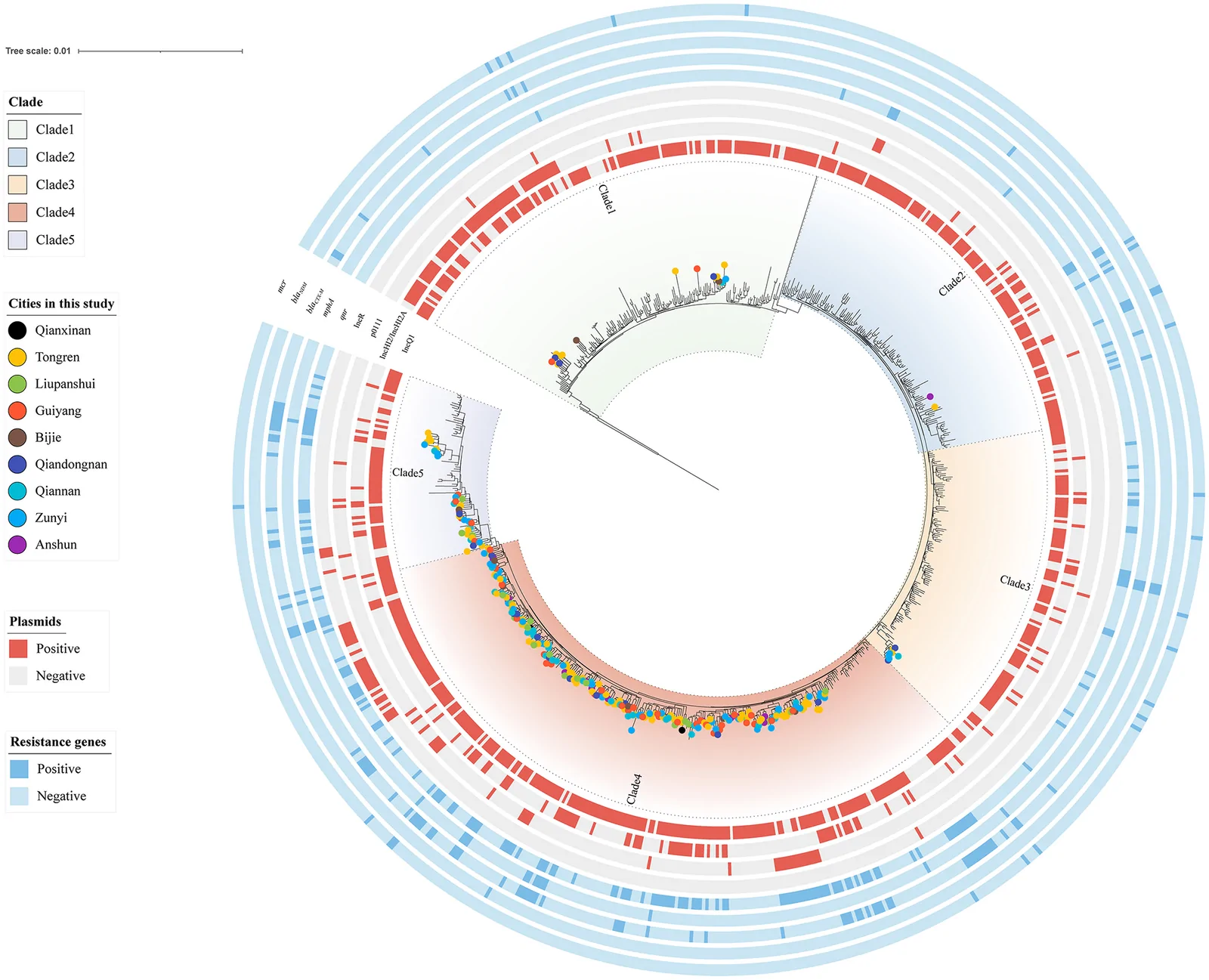
Mapping of CIA-resistant ARGs and plasmid replicons onto the phylogenetic tree of *Salmonella* ST34 isolates. The concentric rings represented the presence or absence of genetic elements: the outer five rings correspond to five types of ARGs, and the inner four rings indicate the four plasmid replicons.

We next mapped the CIA-resistant ARGs and the four most common plasmid incompatibility groups onto the phylogenetic tree of *Salmonella* ST34 (Figure 12). Our analysis revealed a concentration of key ARGs within Clades 4 and 5. The *qnr* and *bla*_*CTX*−*M*_ genes were highly enriched in Clades 4 and 5, accounting for 78.3% (126/161) and 78.4% (76/97) of their respective totals. In contrast, the prevalence of these genes in other clades remained low (< 12.5%). This trend was particularly pronounced among isolates from Guizhou, with 98.1% (104/106) of *qnr*-positive and 95.0% (57/60) of *bla*_*CTX*−*M*_-positive isolates located in Clades 4 and 5. Furthermore, the *mphA* gene was predominantly found in Clade 4, which included 17 out of the 23 *mphA*-positive isolates (73.9%). Additionally, a single isolate from Guizhou carrying the *bla*_*NDM*−5_ gene was identified in Clade 4. In contrast, *mcr* genes were distributed sporadically across all five clades. The two *mcr*-positive isolates identified in this study, which contained *mcr-1.1* and *mcr-9.1*, respectively, were situated in Clades 4 and 5 (Supplementary Data S7).

A similar pattern of distribution was observed for plasmid replicons. The p0111 plasmid was concentrated in Clades 4 and 5, accounting for 82.9% (34/41) of all identified p0111 plasmids. The great majority of these p0111 plasmids in Clades 4 and 5 (97.1%, 33/34) originated from Guizhou Province. Moreover, all isolates from Guizhou that carried IncR plasmids were distributed in these two clades. Notably, IncHI2/IncHI2A plasmids showed a statistically significant association with CIA-resistant ARGs in Clades 4 and 5 (χ^2^ = 112.12, *P* < 0.001), with 93.5% (72/77) of isolates carrying these plasmids co-harboring at least one CIA-resistant ARG.

### 3.9 Distribution of additional MGEs and virulence-associated genes

Screening for integron integrases showed that class 1 integrase (IntI1) was detected in 74 of 281 isolates (26.3%), with prevalence varying across phylogenetic clades (Clade 1: 33.3%, 6/18; Clade 2: 0.0%, 0/2; Clade 3: 25.0%, 2/8; Clade 4: 24.0%, 52/217; Clade 5: 38.9%, 14/36). Additionally, IntI1-positive isolates carried significantly more ARGs than IntI1-negative isolates (mean 16.2 vs. 11.1; Mann-Whitney U test, *P* < 0.001). Class 2 (IntI2) and Class 3 (IntI3) integron integrases were not detected, with the best matches < 58.7% identity to the reference sequences. The IS*6*-family transposase IS*26* was widely distributed (89.7%, 252/281), whereas the IS*1380*-family transposase ISEcp1 was detected in 15 isolates (5.3%) and was confined to Clades 4 and 5 (Supplementary Data S8).

Targeted screening of 34 canonical *Salmonella* virulence loci detected 33 of 34 loci in ≥99.6% of isolates. Mean nucleotide identity exceeded 95% for 32 of these 33 loci. The remaining locus, *invF*, showed a lower mean identity of 86.8%. The plasmid-borne virulence gene *spvC* was not detected in any isolate (Supplementary Data S9). Contig-level analysis across all 281 isolates mapped 9,272 virulence-locus detections to their respective assembly contigs. Of these, 9,271 (99.9%) were located on chromosome-associated contigs. A single detection, *csgD* in isolate SM2022105, was located on a 7.3 kb plasmid-associated contig. This contig carried none of the 12 ARGs annotated in the same isolate (Supplementary Data S10). Across the entire collection, no plasmid-associated contig was found to harbor both an ARG and a virulence-associated gene. The virulence-associated gene panel also showed no appreciable variation in carriage among clades or cgSTs.

### 3.10 Coding capacity and gene content

Gene prediction across the 281 *Salmonella* 1,4,[5],12:i:- ST34 genomes yielded a mean of 4,795.5 predicted protein-coding genes per genome (range: 4,516–5,162; SD = 117.3; coefficient of variation: 2.4%). Using the established 3,002-locus *Salmonella* enterica cgMLST scheme as the core-genome reference, the number of predicted genes outside this reference averaged 1,793 per isolate, representing 37.4% of the total coding capacity (Supplementary Data S11, S12). Gene content varied across phylogenetic clades, with Clades 4 and 5 showing the largest mean gene complements (4,804 and 4,803 genes, respectively) and Clade 3 the smallest (4,717 genes). Among the 10 predominant cgSTs, cgST52428 carried the largest mean gene complement (4,841 genes), whereas cgST227213 carried the smallest (4,584 genes).

## 4 Discussion

*Salmonella* 1,4,[5],12:i:- was first identified in poultry in the late 1980s, and its frequency has increased rapidly over the last 20 years (Elnekave et al., 2018). In our previous research, *Salmonella* 1,4,[5],12:i:- was the top serotype, and the majority of *Salmonella* 1,4,[5],12:i:- isolates belonged to ST34 between 2013 and 2018 in Guizhou Province (Long et al., 2022). Meanwhile, the MDR and XDR were emergent problems within *Salmonella* 1,4,[5],12:i:- isolates. These findings highlight the urgent need for improved antibiotic stewardship and infection control in the region. To further explore the temporal evolution of AMR, the mechanisms underlying these high resistance rates, and the molecular epidemiology, we extended our study to include antibiotic susceptibility testing and whole-genome sequencing of *Salmonella* 1,4,[5],12:i:- isolates from 2019 to 2023 in Guizhou Province. This additional research will help us identify more ARGs and assess the associations between plasmid-associated ARGs and plasmid replicons. We aim to provide comprehensive data that can inform evidence-based policies to reduce the burden of AMR in Guizhou Province.

In this study, we noticed that the resistance rates of *Salmonella* 1,4,[5],12:i:- isolates to first-line antibiotics increased in Guizhou Province. Specifically, 22.4% of isolates were resistant to ciprofloxacin, and 11.4% to azithromycin, representing an obvious increase compared to the rates observed from 2013 to 2018 (Long et al., 2022). This upward trend raises concerns regarding the continued empirical use of these first-line agents for salmonellosis in Guizhou, suggesting that local treatment guidelines may warrant periodic re-evaluation based on contemporary susceptibility data. However, the resistance rates in Guizhou were lower than those reported in Hangzhou, China (Zheng et al., 2024) and Italy (Peruzy et al., 2025). The resistance rates to cefotaxime and ceftazidime were 39.1 and 18.5%, respectively. These rates are markedly lower than those reported in the Guangdong and Zhejiang Provinces of China (Nambiar et al., 2024; Sun et al., 2024), but higher than those observed in Thailand and the United Kingdom (Lopez-Garcia et al., 2023). These findings suggest notable regional variations in AMR. We also observed a rise in the number of MDR and XDR isolates in Guizhou Province. The MDR rate reached 91.9%, with the number of MDR isolates rising each year. The number of XDR isolates doubled from five in 2021 to 10 in 2023. The rapid increase in MDR and XDR isolates is a significant public health concern that requires urgent attention and intervention to prevent further spread and protect the population's health. Meanwhile, we detected that one isolate exhibited resistance to ertapenem and two isolates were resistant to colistin. Carbapenems and colistin are considered last-resort treatments for severe intestinal infections caused by MDR and XDR *Salmonella* (Jean et al., 2022; Liu et al., 2024). The emergence of resistance to these antibiotics poses a significant threat to their clinical effectiveness. In 2022, a narrow AMR pattern was observed, coinciding with COVID-19 control measures in Guizhou. Travel restrictions likely reduced exposure to resistant bacteria, while routine disinfection may have temporarily hindered the horizontal transfer of ARGs. These findings suggest that human interventions limit exposure to resistant bacteria and reduce antibiotic selection pressure, potentially slowing the development of resistant isolates.

A comprehensive study was conducted on the dynamic monitoring of ARGs and point mutations in *Salmonella* 1,4,[5],12:i:- ST34 isolates in Guizhou Province from 2019 to 2023. This study revealed a marked increase in the diversity of ARGs over these 5 years, rising from 41 distinct types in 2019 to 52 in 2023. Of particular concern, several high-threat ARGs emerged during this period, conferring resistance to a wide range of antibiotic classes, including fluoroquinolones (*qnrB4*), macrolides (*mphA* and *mrx*), cephalosporins (*bla*_*TEM*−176_, *bla*_*DHA*−1_), carbapenems (*bla*_*NDM*−5_), and colistin (*mcr-1.1* and *mcr-9.1*). These developments pose considerable challenges to global public health, as the effectiveness of many commonly used antibiotics is being increasingly undermined. The rapid spread of these ARGs among bacterial populations calls for urgent action from healthcare systems, governments, and research institutions worldwide. Enhanced international collaboration and data sharing are vital for monitoring and containing the spread of high-threat ARGs to ensure that we can continue treating infections and effectively protect community health.

Our study analyzed the relationship between ARGs and resistance phenotypes. We observed a strong association for several antibiotics, such as tetracycline, macrolides, 3GCs, carbapenems, and colistin (80.8–100.0% consistency; *Kappa*: 0.50–1.00), which indicated that these ARGs can effectively predict resistance phenotypes. Resistance to carbapenems is mainly mediated by carbapenemases, with New Delhi metallo-β-lactamase (*NDM*) being the most common type in Chinese clinical practice (Wu et al., 2023), hydrolyzing the β-lactam ring to confer resistance (Ramirez-Castillo et al., 2023). In 2015, plasmid-mediated colistin resistance emerged in China and spread via *mcr-1.1* (Liu et al., 2016). Our findings identified an isolate with *mcr-1.1* resistant to colistin in 2021, but an isolate with *mcr-9.1* in 2023 showed no resistance. Research from Italy indicates *mcr-9.1* requires the qseB-C system for resistance (Diaconu et al., 2021). However, this gene is located on IncHI2 plasmids that are often enriched with MGEs and may carry *ESBL* and heavy metal resistance genes. Genetic remodeling or host evolution could potentially convert it into a significant threat. Therefore, continuous monitoring of the genetic context of *mcr-9.1* using WGS is essential to prevent the transformation of this “silent gene” into one that confers clinical resistance. Furthermore, our analyses indicated low consistency between resistance genotypes and phenotypes for sulfonamides and phenicols (*Kappa* < 0.40). Sulfamethoxazole-trimethoprim is a combination of sulfamethoxazole and trimethoprim, and its resistance may involve the coexistence of *sul* and *dfrA* genes. Moreover, the detection of phenotypic resistance without detectable phenicol resistance genes implies the existence of alternative resistance mechanisms (Dawan et al., 2022). This study provides a preliminary elucidation of the relationship between ARGs and phenotypes in *Salmonella* 1,4,[5],12:i:- from Guizhou Province, laying the groundwork for future research into resistance mechanisms.

The acquisition, transmission, and maintenance of ARGs typically depend on specific genetic vectors (Botelho and Schulenburg, 2021). Among these, plasmids are one of the major types of MGEs and play a pivotal role in the horizontal transfer of ARGs among bacteria, serving as critical drivers of cross-host and cross-regional dissemination of AMR (Castaneda-Barba et al., 2024). In this study, we found that ARGs conferring resistance to CIAs were predicted to be plasmid-associated, including 96.7% of *ESBL* genes and all ARGs conferring resistance to macrolides, and colistin. These findings suggest that plasmids may be the primary vectors responsible for resistance to CIAs in *Salmonella* 1,4,[5],12:i:- isolates from Guizhou Province. Additionally, studies have shown that the genome of *Salmonella* 1,4,[5],12:i:- harbors diverse plasmid types, which enhance the bacterial adaptability to hosts and environments (Botelho and Schulenburg, 2021). In the current study, a total of 26 plasmid replicons were identified, with a carriage rate of 96.8%. Meanwhile, there was a marked increase in plasmid replicon types (7 to 21) from 2019 to 2023, highlighting the extensive distribution and diversity of plasmids among *Salmonella* 1,4,[5],12:i:- ST34 isolates in Guizhou Province. Among these plasmids, IncQ1 was the predominant plasmid replicon (84.3%), a trend consistent with reports from Sichuan Province (Zhou et al., 2023) but notably higher than the rates observed in Singapore (Lim et al., 2024). Previous research has indicated a positive association between IncQ1 and the MDR phenotype in *Salmonella* 1,4,[5],12:i:- ST34 (Wu X. et al., 2025), potentially explaining the higher incidence of IncQ1 in certain regions. These indicated that IncQ1 plasmids may contribute to the spread of MDR among *Salmonella* 1,4,[5],12:i:- ST34 isolates, warranting further investigation into their specific roles in AMR dissemination. The carriage rate of IncHI2/IncHI2A was 26.3%, ranking second and comparable to rates in Shaanxi (Shi et al., 2025), Guangdong (Sun et al., 2024), and Sichuan (Li et al., 2025). These large plasmids are major drivers of resistance transmission, often facilitating the conjugative transfer of ARGs (Billman-Jacobe et al., 2018). Correlation analysis demonstrated significant associations between plasmid-associated ARGs (for fluoroquinolones, macrolides, and 3GCs) and IncHI2/IncHI2A plasmids (*P* < 0.05), which is consistent with reports identifying IncHI2/IncHI2A plasmids as the predominant vehicle encoding resistance to CIAs in both ST34 and other *Salmonella* sequence types (Bloomfield et al., 2022; Chen et al., 2016). These results suggest that acquired resistance associated with IncHI2/IncHI2A plasmids may be the key mechanism underlying resistance to CIAs in *Salmonella* 1,4,[5],12:i:- from Guizhou. These findings also have potential implications for antimicrobial drug discovery and therapeutic development. The rising resistance to fluoroquinolones, macrolides, and 3GCs in this study underscores the diminishing utility of these first-line antibiotics and the urgent need for novel antibiotics targeting MDR/XDR *Salmonella* (Jesudason, 2024). Notably, the significant association between IncHI2/IncHI2A plasmids and multiple CIA-resistant ARGs suggests that strategies aimed at disrupting plasmid maintenance or inhibiting conjugative transfer could serve as adjunctive therapeutic approaches to limit resistance dissemination (Touati et al., 2026). Furthermore, the detection of genotypically present but phenotypically unexpressed determinants such as *mcr-9.1* highlights the value of prospective genomic surveillance in guiding early-stage drug discovery; confirming whether such genes are truly transcriptionally or functionally silent would require dedicated expression or functional assays, which were beyond the scope of this study, but their presence alone warrants continued monitoring given the potential for future activation to compromise last-resort antibiotics (Song et al., 2024). Collectively, these observations support the integration of genomic epidemiology into antimicrobial stewardship programs to inform empirical treatment guidelines and prioritize targets for drug development.

Molecular epidemiological studies of *Salmonella* 1,4,[5],12:i:- are essential for preventing and controlling its transmission. Among commonly used molecular tools, cgMLST and cgSNP are frequently applied for traceability analysis of *Salmonella* serotypes with high genetic similarity (Van Puyvelde et al., 2019; Zhang et al., 2024). In this study, 281 *Salmonella* 1,4,[5],12:i:- ST34 isolates were divided into 37 cgSTs, with cgST52428 being the most common. Additionally, cgST52428 was also the most prevalent among isolates resistant to fluoroquinolones, azithromycin, and 3GCs, and harbored *qnrS1, mphA, bla*_*CTX*−*M*_, and IncHI2/IncHI2A plasmids most frequently, suggesting that its local dominance may be driven by an enhanced capacity to acquire and maintain resistance determinants. The predominance of cgST52428 is not limited to Guizhou; a recent study from Jeollanam-do, South Korea, similarly identified this cgST as the predominant cgST among ST34 isolates (23/24, 95.8%) (Go et al., 2025). Despite differences in sample size and isolate sources between the two studies, the consistent dominance of this clone across East Asian regions suggests that it may possess enhanced fitness and transmissibility, enabling its successful regional dissemination. These findings underscore the need for cross-border genomic surveillance to monitor the spread of this potentially high-risk clone. MST analysis indicated that 88.6% of isolates from Guizhou clustered within A3 and A4, exhibiting short allele distances to isolates from other Chinese provinces and municipalities. This suggests that transmission of *Salmonella* 1,4,[5],12:i:- in Guizhou may occur through both intra- and inter-provincial spread within China. Overall, the research highlights the genetic relatedness of *Salmonella* ST34 isolates from Guizhou with domestic and international isolates, emphasizing the significance of regional and global genetic connections.

The cgSNP analysis provided insights into the evolutionary relationships of *Salmonella* 1,4,[5],12:i:- ST34 in Guizhou through three dimensions: isolation time, location, and source. When comparing ST34 *Salmonella* Typhimurium and *Salmonella* 1,4,[5],12:i:-, all *Salmonella* Typhimurium ST34 isolates clustered within Clade 1, which was phylogenetically closest to the reference strain LT2. The temporal analysis revealed that *Salmonella* 1,4,[5],12:i:- ST34 isolates were obtained later than the *Salmonella* Typhimurium ST34 isolates. These results suggest that *Salmonella* 1,4,[5],12:i:- may have evolved from *Salmonella* Typhimurium ST34, a finding that aligns with domestic studies (Wang et al., 2023a). Molecular traceback analysis demonstrated close genetic relationships between the isolates from Guizhou and those from Sichuan and Guangdong Provinces, with fewer than 160 SNP differences. Sichuan, being geographically adjacent to Guizhou, shares similar climate conditions and dietary customs, which likely contribute to the high genetic similarity of their *Salmonella* 1,4,[5],12:i:- isolates. These findings highlight the potential value of establishing a coordinated regional surveillance network with neighboring provinces to enhance early detection and containment of high-risk clones across provincial boundaries. Additionally, Guangdong serves as a national distribution hub within a well-developed logistics network and extensive trade connections, underscoring the need for surveillance to extend beyond geographic proximity to include major trade-linked dissemination routes.

Some isolates from Guizhou demonstrated close genetic relationships with isolates from South Korea, Europe, and the United States. These may suggest that, due to global trade and frequent international travel, foodborne pathogens could be transmitted across borders through global food supply chains or human movement. This may suggest that, due to global trade and frequent international travel, foodborne pathogens could be transmitted across borders through global food supply chains or human movement, highlighting the importance of global genomic surveillance of pathogens and the need for standardized detection protocols. Additionally, pork-derived isolates exhibited the shortest genetic distances to human-derived *Salmonella* 1,4,[5],12:i:- isolates in Guizhou, consistent with earlier reports from the European Center for Disease Prevention and Control (ECDC) and the European Food Safety Authority (EFSA), which have suggested that pork is a major source of human salmonellosis (Ferrari et al., 2019; European Food Safety Authority (EFSA) and European Centre for Disease Prevention and Control (ECDC), 2018). This epidemiological link is further substantiated by a recent comparative genomic study from Sichuan Province, the largest pork-production region in China, which not only identified ST34 as one of the predominant sequence types shared between pork and human isolates but also demonstrated a higher burden of ARGs in pork-derived strains, particularly those conferring resistance to tetracycline, chloramphenicol, and sulfonamides, reflecting the selective pressure from antibiotic use in animal husbandry (Zuo et al., 2025). Pork, being a commodity commonly associated with a high risk of *Salmonella* contamination, is a significant source of human salmonellosis (Bonardi, 2017; Wilson et al., 2020). Given that China is one of the world's largest consumers of pork, it is crucial to establish a comprehensive prevention and control system that encompasses breeding, slaughtering, and consumption. Efforts should focus on improving rapid screening technologies during slaughter, formulating standards for cold-chain transportation, and implementing regular surveillance at retail markets. These measures will more effectively prevent the transmission of foodborne pathogens to humans through meat products.

Phylogenetic analysis revealed clustering of CIA-resistant ARGs alongside the four most common plasmid replicons within Clades 4 and 5. These two clades exhibited greater genetic distance from the LT2 strain compared to other clades and were predominantly composed of isolates collected from 2019 onward (90.6%). Additionally, the majority of isolates from Guizhou were found within these clades. Almost all isolates from Guizhou carrying the *qnr, bla*_*CTX*−*M*_, *mphA, bla*_*NDM*_, and *mcr* genes were situated in these two clades. This spatiotemporal and evolutionary relationship may indicate that *Salmonella* 1,4,[5],12:i:- ST34 in this region is evolving toward lineages with a high burden of CIA-resistant determinants, likely influenced by antimicrobial selection pressure. Notably, we observed a significant association between IncHI2/IncHI2A plasmids and CIA-resistant ARGs in Clades 4 and 5 (χ^2^ = 112.12, *P* < 0.001), with 93.5% (72/77) of IncHI2/IncHI2A-positive isolates carrying at least one ARG. These findings emphasize the urgent need to prioritize surveillance of Clades 4, 5 and IncHI2/IncHI2A plasmids and to implement antimicrobial stewardship programs to mitigate further selection. Future research should focus on elucidating the precise mechanisms by which these plasmids facilitate the expression of CIA-resistant genes. Such insights will be crucial for developing targeted public health strategies against the evolving *Salmonella* 1,4,[5],12:i:- isolates in Guizhou Province.

Beyond plasmids, class 1 integrons and specific insertion sequences appear to contribute to ARG accumulation in this population. IntI1-positive isolates carried significantly more ARGs than *intI1*-negative isolates (*P* < 0.001), consistent with the role of class 1 integrons in capturing and disseminating ARG cassettes in Enterobacteriaceae (Wang T. et al., 2023). ISEcp1 was detected only in Clades 4 and 5, where *bla*_*CTX*−*M*_ and other CIA-resistant ARGs were also enriched, consistent with the role of ISEcp1 in mobilizing *bla*_*CTX*−*M*_ genes (Yang et al., 2024). Class 2 and class 3 integron integrases were not detected using the thresholds applied here, which may reflect true absence or sequence divergence beyond our detection limit. Taken together, these findings suggest that multiple MGEs may contribute to ARG dissemination in *Salmonella* 1,4,[5],12:i:-, but short-read data cannot resolve their complete structures or linkages. Long-read sequencing would be needed to characterize the full architecture of integrons, the insertion sites of IS elements, and their relationships with ARGs on both plasmids and chromosomes.

Targeted screening of 34 canonical virulence loci showed that the virulence-associated gene was broadly conserved in *Salmonella* 1,4,[5],12:i:- from Guizhou, with most loci detected in nearly all isolates. The plasmid-borne virulence gene *spvC* was not detected, and contig-level analysis found no co-localization of ARGs and virulence loci on the same plasmid-associated contig. This pattern may support a model in which core virulence determinants form a stable, chromosomally encoded backbone, while AMR is acquired dynamically through horizontal transfer of MGEs s superimposed on this backbone. Gene-content analysis provided additional context. Using the 3,002-locus cgMLST scheme as a reference, the *Salmonella* 1,4,[5],12:i:- ST34 genome comprised a conserved backbone and a variable component that differed by phylogenetic clade and cgST. The predominant cgST52428 carried the largest mean gene complement among the top 10 cgSTs, consistent with the possibility that gene accumulation (potentially including AMR- and mobility-associated elements) contributes to its epidemiological success. However, the gene-content estimate relies on total predicted gene counts relative to a fixed core-genome reference rather than whole-genome ortholog clustering (e.g., Roary or Panaroo) due to computational resource constraints; it approximates coding capacity rather than providing a formal core, accessory, or unique gene classification. A complete pan-genome analysis with *de novo* ortholog clustering remains an important direction for future work.

While our study presents a comprehensive analysis, it is essential to acknowledge several important limitations. First, our analysis only focused on human-derived isolates, which may not fully represent the presence in other sources, such as food, animal, and environmental sources. To effectively combat the threats posed by *Salmonella* 1,4,[5],12:i:-, future research should aim to include a more diverse range of sample sources to overcome the limitation and provide a more complete understanding of the subject matter. Second, the utilization of long-read sequencing technologies accelerates the assembly of more comprehensive *Salmonella* genomes, thereby facilitating the precise localization of ARGs and virulence genes within specific plasmid replicons and the reconstruction of complete integron and insertion sequence structures, enabling a deeper investigation of the genetic basis of AMR and virulence. However, due to resource constraints, we were unable to employ long-read sequencing in this study, which may have limited the depth of our genomic analysis. Future studies should consider incorporating long-read sequencing to enhance the resolution and accuracy of genomic investigations. By addressing these limitations in future research, we can further enhance our understanding of *Salmonella* 1,4,[5],12:i:- and develop more effective strategies to prevent and control its spread.

## 5 Conclusion

This study systematically characterized the genomic profiles of *Salmonella* 1,4,[5],12:i:- ST34 in Guizhou Province by analyzing isolates from patients with diarrhea. Our findings reveal a rapid escalation in AMR and plasmid diversity in *Salmonella* 1,4,[5],12:i:- ST34 in Guizhou. The MDR rate reached 91.9%, with XDR isolate counts doubling from 2021 to 2023. Additionally, the diversity of ARGs increased from 41 types in 2019 to 52 in 2023, and the variety of plasmid replicon types also expanded from 7 to 21. Importantly, high-threat ARGs (such as *bla*_*CTX*−*M*_ variants, *mcr* variants, and *qnr* family) have been identified, with the majority predicted to be plasmid-associated. Class 1 integrons carriage was associated with higher ARG burdens, while the virulence-associated genes were broadly conserved and predominantly chromosome-associated. This highlights the urgent need to strengthen surveillance and early warning systems for plasmid-associated resistance in *Salmonella* 1,4,[5],12:i:- ST34. Moreover, molecular epidemiological analysis elucidated the genetic relationships of *Salmonella* ST34 isolates. Isolates from Guizhou primarily clustered together and exhibited the highest genetic similarity to pork-derived isolates, suggesting a potential transmission pathway and highlighting the need to investigate foodborne transmission routes. Phylogenetic analysis revealed that Guizhou isolates mainly clustered in specific clades enriched with CIA-resistant ARGs and plasmid replicons, further underscoring the critical role of plasmid-associated antibiotic resistance in the dissemination and evolution of *Salmonella* 1,4,[5],12:i:- ST34 in this region.

## Data Availability

The datasets presented in this study can be found in online repositories. The names of the repository/repositories and accession number(s) can be found in the article/Supplementary material.

## References

[B1] Bai G. You L. Long L. Wang D. Wang M. Wang J. . (2022). The CRISPR genotypes and genetic diversity of different serogroups of nontyphoidal *Salmonella* in Guizhou Province, 2013–2018. PLoS ONE. 17:e0278321. doi: 10.1371/journal.pone.0278321 36520925 PMC9754226

[B2] Billman-Jacobe H. Liu Y. Haites R. Weaver T. Robinson L. Marenda M. . (2018). PSTM6-275, a conjugative IncHI2 plasmid of *Salmonella* enterica that confers antibiotic and heavy-metal resistance under changing physiological conditions. Antimicrob. Agents. Chemother. 62:e02357–17. doi: 10.1128/AAC.02357-17 29439975 PMC5923156

[B3] Bloomfield S. Duong V. T. Tuyen H. T. Campbell J. I. Thomson N. R. Parkhill J. . (2022). Mobility of antimicrobial resistance across serovars and disease presentations in non-typhoidal *Salmonella* from animals and humans in Vietnam. Microb. Genom. 8:mgen000798. doi: 10.1099/mgen.0.000798 35511231 PMC9465066

[B4] Bonardi S. (2017). *Salmonella* in the pork production chain and its impact on human health in the European Union. Epidemiol. Infect. 145, 1513–26. doi: 10.1017/S095026881700036X 28241896 PMC9203350

[B5] Botelho J. Schulenburg H. (2021). The role of integrative and conjugative elements in antibiotic resistance evolution. Trends Microbiol. 29, 8–18. doi: 10.1016/j.tim.2020.05.011 32536522

[B6] Castaneda-Barba S. Top E. M. Stalder T. (2024). Plasmids, a molecular cornerstone of antimicrobial resistance in the One Health era. Nat. Rev. Microbiol. 22, 18–32. doi: 10.1038/s41579-023-00926-x 37430173 PMC12440250

[B7] Chen W. Fang T. Zhou X. Zhang D. Shi X. Shi C. (2016). IncHI2 plasmids are predominant in antibiotic-resistant *Salmonella* isolates. Front. Microbiol. 7:1566. doi: 10.3389/fmicb.2016.01566 27746775 PMC5043248

[B8] Chung The H. Pham P. Ha Thanh T. Phuong L. V. K. Yen N. P. Le S. H.. (2023). Multidrug resistance plasmids underlie clonal expansions and international spread of *Salmonella* enterica serotype 1,4,[5],12:i:- ST34 in southeast Asia. Commun. Biol. 6:1007. doi: 10.1038/s42003-023-05365-1 37789208 PMC10547704

[B9] Clark C. G. Landgraff C. Robertson J. Pollari F. Parker S. Nadon C. . (2020). Distribution of heavy metal resistance elements in Canadian *Salmonella* 4,[5],12:i:- populations and association with the monophasic genotypes and phenotype. PLoS ONE. 15:e0236436. doi: 10.1371/journal.pone.0236436 32716946 PMC7384650

[B10] CLSI (2023). Performance Standards for Antimicrobial Susceptibility Testing. CLSI Supplement M100, 33rd Edn. Wayne, PA: Clinical and Laboratory Standards Institute.

[B11] Cuypers W. L. Meysman P. Weill F. X. Hendriksen R. S. Beyene G. Wain J. . (2023). A global genomic analysis of *Salmonella* concord reveals lineages with high antimicrobial resistance in Ethiopia. Nat. Commun. 14:3517. doi: 10.1038/s41467-023-38902-x 37316492 PMC10267216

[B12] Dawan J. Li Y. Lu F. He X. Ahn J. (2022). Role of efflux pump-mediated antibiotic resistance in quorum sensing-regulated biofilm formation by *Salmonella* Typhimurium. Pathogens. 11:147. doi: 10.3390/pathogens11020147 35215091 PMC8877114

[B13] Diaconu E. L. Alba P. Feltrin F. Di Matteo P. Iurescia M. Chelli E. . (2021). Emergence of IncHI2 plasmids with mobilized colistin resistance *mcr-9* gene in *ESBL*-producing, multidrug-resistant *Salmonella* Typhimurium and its monophasic variant ST34 from food-producing animals in Italy. Front. Microbiol. 12:705230. doi: 10.3389/fmicb.2021.705230 34335538 PMC8322855

[B14] Elnekave E. Hong S. Mather A. E. Boxrud D. Taylor A. J. Lappi V. . (2018). *Salmonella* enterica serotype 4,[5],12:i:- in swine in the United States Midwest: an emerging multidrug-resistant clade. Clin. Infect. Dis. 66, 877–85. doi: 10.1093/cid/cix909 29069323

[B15] European Food Safety Authority (EFSA) and European Centre for Disease Prevention and Control (ECDC) (2023). The European Union one health 2022 zoonoses report. EFSA J. 21:e8442. doi: 10.2903/j.efsa.2023.8442 38089471 PMC10714251

[B16] European Food Safety Authority (EFSA) and European Centre for Disease Prevention and Control (ECDC) (2019). The European Union summary report on antimicrobial resistance in zoonotic and indicator bacteria from humans, animals and food in 2017. EFSA J. 17:e5598. doi: 10.2903/j.efsa.2019.5598 PMC7009238 32626224

[B17] European Food Safety Authority (EFSA) and European Centre for Disease Prevention and Control (ECDC) (2018). The European Union summary report on trends and sources of zoonoses, zoonotic agents and food-borne outbreaks in 2017. EFSA J. 16:e5500. doi: 10.2903/j.efsa.2018.5500 PMC7009540 32625785

[B18] Ferrari R. G. Rosario D. K. A. Cunha-Neto A. Mano S. B. Figueiredo E. E. S. Conte-Junior C. A. (2019). Worldwide epidemiology of *Salmonella* serovars in animal-based foods: a meta-analysis. Appl. Environ. Microbiol. 85:e00591–19. doi: 10.1128/AEM.00591-19 31053586 PMC6606869

[B19] Garcia P. Malorny B. Rodicio M. R. Stephan R. Hachler H. Guerra B. . (2016). Horizontal acquisition of a multidrug-resistance module (R-type ASSuT) is responsible for the monophasic phenotype in a widespread clone of *Salmonella* serovar 4,[5],12:i. Front. Microbiol. 7:680. doi: 10.3389/fmicb.2016.00680 27242707 PMC4861720

[B20] Garcia-Soto S. Tomaso H. Linde J. Methner U. (2021). Epidemiological analysis of *Salmonella* enterica subsp. Enterica serovar Dublin in German cattle herds using whole-genome sequencing. Microbiol. Spectr. 9:e0033221. doi: 10.1128/Spectrum.00332-21 34523945 PMC8557873

[B21] Go E. Kang B. R. Na H. Y. Lim H. W. Yang H. L. Shin M. Y. . (2025). Genomic insights into antimicrobial resistance and virulence of monophasic *Salmonella enterica* I 4,[5],12:i:- isolates from clinical and environmental sources in Jeollanam-do, Korea. Microorganisms. 13:2729. doi: 10.3390/microorganisms13122729 41471932 PMC12735187

[B22] Guibourdenche M. Roggentin P. Mikoleit M. Fields P. I. Bockemühl J. Grimont P. A. D. . (2010). Supplement 2003-2007 (no. 47) to the white-kauffmann-le minor scheme. Res. Microbiol. 161, 26–9. doi: 10.1016/j.resmic.2009.10.002 19840847

[B23] Jean S. O. S. Harnod D. Hsueh P. R. (2022). Global threat of carbapenem-resistant gram-negative bacteria. Front. Cell. Infect. Microbiol. 12:823684. doi: 10.3389/fcimb.2022.823684 35372099 PMC8965008

[B24] Jesudason T. (2024). WHO publishes updated list of bacterial priority pathogens. Lancet Microbe. 5:100940. doi: 10.1016/j.lanmic.2024.07.003 39079540

[B25] Larkin L. Pardos de la Gandara M. Hoban A. Pulford C. Jourdan-Da Silva N. de Valk H. . (2022). Investigation of an international outbreak of multidrug-resistant monophasic *Salmonella* Typhimurium associated with chocolate products, EU/EEA and United Kingdom, February to April 2022. Eurosurveillance. 27:2200314. doi: 10.2807/1560-7917.ES.2022.27.15.2200314 35426359 PMC9012091

[B26] Li C. Jiang X. Yang T. Ju Y. Yin Z. Yue L. . (2022). Genomic epidemiology of carbapenemase-producing klebsiella pneumoniae in China. Genom. Proteomics Bioinformatics. 20, 1154–67. doi: 10.1016/j.gpb.2022.02.005 PMC10225488 35307590

[B27] Li L. Sun H. Zhao J. Sheng H. Li M. Zhao L. . (2025). The genomic characteristics of dominant *Salmonella* enterica serovars from retail pork in Sichuan Province, China. Int. J. Food Microbiol. 434:111129. doi: 10.1016/j.ijfoodmicro.2025.111129 40024181

[B28] Lim Y. C. Ong K. H. Khor W. C. Chua F. Y. X. Lim J. Q. Tan L. K. . (2024). Sequence types and antimicrobial resistance profiles of *Salmonella* Typhimurium in the food chain in Singapore. Microorganisms. 12:1912. doi: 10.3390/microorganisms12091912 39338586 PMC11434088

[B29] Liu J. H. Liu Y. Y. Shen Y. B. Yang J. Walsh T. R. Wang Y. . (2024). Plasmid-mediated colistin-resistance genes: *mcr*. Trends Microbiol. 32, 365–78. doi: 10.1016/j.tim.2023.10.006 38008597

[B30] Liu Y. Y. Wang Y. Walsh T. R. Yi L. X. Zhang R. Spencer J. . (2016). Emergence of plasmid-mediated colistin resistance mechanism *mcr-1* in animals and human beings in China: a microbiological and molecular biological study. Lancet Infect. Dis. 16, 161–8. doi: 10.1016/S1473-3099(15)00424-7 26603172

[B31] Long L. You L. Wang D. Wang M. Wang J. Bai G. . (2022). Highly prevalent MDR, frequently carrying virulence genes and antimicrobial resistance genes in *Salmonella* enterica serovar 4,[5],12:i:- isolates from Guizhou Province, China. PLoS ONE. 17:e0266443. doi: 10.1371/journal.pone.0266443 35588421 PMC9119451

[B32] Lopez-Garcia A. V. AbuOun M. Nunez-Garcia J. Nale J. Y. Gaylov E. E. Phothaworn P. . (2023). Pathogen genomics and phage-based solutions for accurately identifying and controlling *Salmonella* pathogens. Front. Microbiol. 14:1166615. doi: 10.3389/fmicb.2023.1166615 37234523 PMC10206635

[B33] Magiorakos A. P. Srinivasan A. Carey R. B. Carmeli Y. Falagas M. E. Giske C. G. . (2012). Multidrug-resistant, extensively drug-resistant and pandrug-resistant bacteria: an international expert proposal for interim standard definitions for acquired resistance. Clin. Microbiol. Infect. 18, 268–81. doi: 10.1111/j.1469-0691.2011.03570.x 21793988

[B34] Marder Mph E. P. Griffin P. M. Cieslak P. R. Dunn J. Hurd S. Jervis R. . (2018). Preliminary incidence and trends of infections with pathogens transmitted commonly through food-foodborne diseases active surveillance network, 10 United States Sites, 2006–2017. MMWR. Morb. Mortal. Wkly Rep. 67, 324-8. doi: 10.15585/mmwr.mm6711a3 PMC5868202 29565841

[B35] Nambiar R. B. Elbediwi M. Ed-Dra A. Wu B. Yue M. (2024). Epidemiology and antimicrobial resistance of *Salmonella* serovars Typhimurium and 4,[5],12:i- recovered from hospitalized patients in China. Microbiol. Res. 282:127631. doi: 10.1016/j.micres.2024.127631 38330818

[B36] Peruzy M. F. Murru N. Carullo M. R. La Tela I. Rippa A. Balestrieri A. . (2025). Antibiotic-resistant *Salmonella* circulation in the human population in Campania region (2010–2023). *Antibiotics-Basel*. 14:189. doi: 10.3390/antibiotics14020189 PMC11851370 40001432

[B37] Ramirez-Castillo F. Y. Guerrero-Barrera A. L. Avelar-Gonzalez F. J. (2023). An overview of carbapenem-resistant organisms from food-producing animals, seafood, aquaculture, companion animals, and wildlife. Front. Vet. Sci. 10:1158588. doi: 10.3389/fvets.2023.1158588 37397005 PMC10311504

[B38] Shi Y. Liu Y. Li S. Wu S. Ma G. Luan Y. . (2025). Genomic characteristics and antibiotic resistance profiles of monophasic *Salmonella* Typhimurium in Shaanxi Province, China. Front. Microbiol. 16:1565631. doi: 10.3389/fmicb.2025.1565631 40291802 PMC12021821

[B39] Song K. Jin L. Cai M. Wang Q. Wu X. Wang S. . (2024). Decoding the origins, spread, and global risks of *mcr-9* gene. EBioMedicine 108:105326. doi: 10.1016/j.ebiom.2024.105326 39260038 PMC11416231

[B40] Sun H. Wan Y. Du P. Bai L. (2020). The epidemiology of monophasic *Salmonella* Typhimurium. Foodborne Pathog. Dis. 17, 87–97. doi: 10.1089/fpd.2019.2676 31532231

[B41] Sun R.-Y. Fang L.-X. Dai J.-J. Chen K.-C. Ke B.-X. Sun J. . (2024). Antimicrobial resistance and population genomics of emerging multidrug-resistant *Salmonella* 4,[5],12:i:- in Guangdong, China. Msystems. 9:e0116423. doi: 10.1128/msystems.01164-23 38747582 PMC11237462

[B42] Tennant S. M. Diallo S. Levy H. Livio S. Sow S. O. Tapia M. . (2010). Identification by PCR of non-typhoidal *Salmonella* enterica serovars associated with invasive infections among febrile patients in Mali. Plos Neglect. Trop. Dis. 4:e621. doi: 10.1371/journal.pntd.0000621 PMC2834738 20231882

[B43] Tian R. Zhou J. Imanian B. (2024). PlasmidHunter: accurate and fast prediction of plasmid sequences using gene content profile and machine learning. Brief. Bioinform. 25:bbae322. doi: 10.1093/bib/bbae322 38960405 PMC11770376

[B44] Touati A. Boufahja F. Touaitia R. Khezami L. Idres T. Grenni P. (2026). Inhibiting horizontal gene transfer to contain antimicrobial resistance: conjugation and plasmid maintenance as druggable targets. Expert Rev. Anti Infect. Ther. 24, 603–25. doi: 10.1080/14787210.2026.2683605 42217234

[B45] Van Puyvelde S. Pickard D. Vandelannoote K. Heinz E. Barbé B. de Block T. . (2019). An African *Salmonella* Typhimurium ST313 sublineage with extensive drug-resistance and signatures of host adaptation. Nat. Commun. 10:4280. doi: 10.1038/s41467-019-11844-z 31537784 PMC6753159

[B46] Wang T. Zhu Y. Zhu W. Cao M. Wei Q. (2023). Molecular characterization of class 1 integrons in carbapenem-resistant Enterobacterales isolates. Microb. Pathog. 177:106051. doi: 10.1016/j.micpath.2023.106051 36858185

[B47] Wang Y. Liu Y. Lyu N. Li Z. Ma S. Cao D. . (2023). The temporal dynamics of antimicrobial-resistant *Salmonella enterica* and predominant serovars in China. Natl. Sci. Rev. 10:nwac269. doi: 10.1093/nsr/nwac269 37035020 PMC10076184

[B48] Wang Z. Gu C. Sun L. Zhao F. Fu Y. Di L. . (2022). Development of a novel core genome MLST scheme for tracing multidrug resistant staphylococcus capitis. Nat. Commun. 13:4254. doi: 10.1038/s41467-022-31908-x 35869070 PMC9307846

[B49] Wang Z. Gu D. Hong Y. Hu Y. Gu J. Tang Y. . (2023a). Microevolution of Salmonella 4,[5],12:i:- derived from Salmonella enterica serovar Typhimurium through complicated transpositions. Cell Rep. 42:113227. doi: 10.1016/j.celrep.2023.113227 37837619

[B50] Wang Z. Jiang Y. Xu H. Jiao X. Wang J. Li Q. (2023b). Poultry production as the main reservoir of ciprofloxacin- and tigecycline-resistant extended-spectrum beta-lactamase (ESBL)-producing *Salmonella enterica serovar* kentucky ST198.2-2 causing human infections in China. Appl. Environ. Microbiol. 89:e0094423. doi: 10.1128/aem.00944-23 37610223 PMC10537671

[B51] Wang Z. Jiang Z. Xu H. Jiao X. Li Q. (2023c). Prevalence and molecular characterization of mcr-1-positive foodborne ST34-Salmonella isolates in China. Microbiol. Res. 274:127441. doi: 10.1016/j.micres.2023.127441 37356255

[B52] Wei X. Long L. You L. Wang M. Wang D. Liu C. . (2023). Serotype distribution, trend of multidrug resistance and prevalence of beta-lactamase resistance genes in human *Salmonella* isolates from clinical specimens in Guizhou, China. PLoS ONE. 18:e0282254. doi: 10.1371/journal.pone.0282254 37079574 PMC10118202

[B53] Wilson C. N. Pulford C. V. Akoko J. Perez Sepulveda B. Predeus A. V. Bevington J. . (2020). *Salmonella* identified in pigs in Kenya and Malawi reveals the potential for zoonotic transmission in emerging pork markets. Plos Neglect. Trop. Dis. 14:e0008796. doi: 10.1371/journal.pntd.0008796 PMC7748489 33232324

[B54] World Health Organization (2024). WHO List of Medically Important Antimicrobials: a Risk Management Tool for Mitigating Antimicrobial Resistance Due to Non-Human Use (7th Revision). Geneva: World Health Organization.

[B55] Wu B. Ed-Dra A. Pan H. Dong C. Jia C. Yue M. (2021). Genomic investigation of *Salmonella* isolates recovered from a pig slaughtering process in Hangzhou, China. Front. Microbiol. 12:704636. doi: 10.3389/fmicb.2021.704636 34305874 PMC8298193

[B56] Wu J. Wen Y. You L. Wei X. Wang J. Zhu G. . (2025). Genome analysis of colistin-resistant *Salmonella* isolates from human sources in Guizhou of southwestern China, 2019–2023. Front. Microbiol. 16:1498995. doi: 10.3389/fmicb.2025.1498995 39944652 PMC11813891

[B57] Wu X. Du J. Zhou X. Peng X. Jia C. Wang B. . (2025). Genomic epidemiology and public health implications of zoonotic monophasic Salmonella Typhimurium ST34. Front. Cell. Infect. Microbiol. 15:1490183. doi: 10.3389/fcimb.2025.1490183 40134787 PMC11933091

[B58] Wu Y. Jiang T. Bao D. Yue M. Jia H. Wu J. . (2023). Global population structure and genomic surveillance framework of carbapenem-resistant *Salmonella* enterica. Drug Resist. Update. 68:100953. doi: 10.1016/j.drup.2023.100953 36841133

[B59] Xiao Y. Wu Y. Zhao W. Cui X. Li M. Xu X. (2022). Genomic characterization of human-derived *Salmonella* in Chengdu. Chin. J. Zoonoses. 38, 433–40. doi: 10.3969/j.issn.1002-2694.2022.05.007

[B60] Xie M. Chen K. Chan E. W. C. Chen S. (2022). Identification and genetic characterization of two conjugative plasmids that confer azithromycin resistance in *Salmonella*. Emerg. Microbes Infect. 11, 1049–57. doi: 10.1080/22221751.2022.2058420 35333699 PMC9009942

[B61] Yang S. Fan J. Yu L. He J. Zhang L. Yu Y. . (2024). Dissemination of ceftriaxone-resistant *Salmonella* Enteritidis harboring plasmids encoding *bla*_*CTX*−*M*−55_ or *bla*_*CTX*−*M*−14_ gene in China. Antibiotics. 13:456. doi: 10.3390/antibiotics13050456 38786182 PMC11117602

[B62] Zeng S. Huang Y. Zhang X. Fu L. Sun Z. Li X. (2023). Molecular characterization of IncFII plasmid carrying *bla*_*NDM*−5_ in a *Salmonella* enterica serovar Typhimurium ST34 clinical isolate in China. Msphere. 8:e0048023. doi: 10.1128/msphere.00480-23 37909767 PMC10732066

[B63] Zhang K. Wang Z. Wang P. Xu H. Jiao X. Li Q. (2024). Prevalence and genetic characteristics of *Salmonella* enterica serovar Meleagridis from animals and humans. Vet. Microbiol. 290:109993. doi: 10.1016/j.vetmic.2024.109993 38278043

[B64] Zhao Q. Y. Zhang L. Yang J. T. Wei H. J. Zhang Y. H. Wang J. Y. . (2024). Diversity of evolution in MDR monophasic *S*. Typhimurium among food animals and food products in Southern China from 2011 to 2018. Int. J. Food Microbiol. 412:110572. doi: 10.1016/j.ijfoodmicro.2024.110572 38237416

[B65] Zheng Z. Yu H. Zheng W. Zhao P. Lou X. Zeng L. . (2024). Antimicrobial resistance characteristics and genomic analysis of *Salmonella* Typhimurium and its monophasic variant in Hangzhou. China. J. Microbiol. Immunol. 44, 1037–47.

[B66] Zhou L. Zhang T. J. Zhang W. Xie C. Yang Y. Chen X. . (2023). Prevalence and genetic diversity of multidrug-resistant *Salmonella* Typhimurium monophasic variant in a swine farm from China. Front. Microbiol. 14:1200088. doi: 10.3389/fmicb.2023.1200088 37396383 PMC10311412

[B67] Zuo H. Yang Y. Su M. Huang W. Wang J. Lei G. . (2025). Comparative genomic and antimicrobial resistance profiles of *Salmonella* strains isolated from pork and human sources in Sichuan, China. Front. Microbiol. 16:1515576. doi: 10.3389/fmicb.2025.1515576 40099182 PMC11911478

