# Supplementary material for: Genomic surveillance reveals escalating antimicrobial resistance and plasmid diversity in clinical Salmonella 1,4,[5],12:i:- ST34 isolates from Guizhou Province, China
Source: Front Microbiol. 2026 Aug 28;17:1875505. doi: 10.3389/fmicb.2026.1875505 (PMC13561910; doi:10.3389/fmicb.2026.1875505)
Supplement: Supplementary file 1 [file Data_Sheet_1.docx]

Supplementary Figures

**Figure S1. Identification of *Salmonella* Typhimurium and *Salmonella* 1,4,[5],12:i:- isolates by mPCR.** Lane M: DL2,000 DNA Marker. Lanes ST: the representative *Salmonella* Typhimurium isolates produced two amplicons (1,000 bp and 1,389 bp). Lanes 3-19: the representative *Salmonella* 1,4,[5],12:i:- isolates produced a single amplicon (1,000 bp). Neg: the negative control (template without DNA).

**Full uncropped gels images of 284 *Salmonella* 1,4,[5],12:i:- isolates in this study**

**Figure S2.** **Identification of *Salmonella* 1,4,[5],12:i:- isolates in this study** **(SM2019002-SM2020086).**

Upper row (Lanes 3-19): SM2019002-SM2019048

Lower row (Lanes 3-19): SM2019050-SM2020086

**Figure S3.** **Identification of *Salmonella* 1,4,[5],12:i:- isolates in this study (SM2020095-SM2021055).**

Upper row (Lanes 2-13): SM2020095-SM2021029

Lower row (Lanes 2-11): SM2021031-SM2021055

**Figure S4.** **Identification of *Salmonella* 1,4,[5],12:i:- isolates in this study (SM2021057-SM2021205).**

Upper row (Lanes 3-19): SM2021057-SM2021126

Lower row (Lanes 3-19): SM2021127-SM2021205

**Figure S5.** **Identification of *Salmonella* 1,4,[5],12:i:- isolates in this study (SM2021207-SM2022036).**

Upper row (Lanes 3-14): SM2021207-SM2021240

Lower row (Lanes 3-14): SM2022004-SM2022036

**Figure S6.** **Identification of *Salmonella* 1,4,[5],12:i:- isolates in this study (SM2022038-SM2022148).**

Upper row (Lanes 3-19): SM2022038-SM2022078

Lower row (Lanes 3-19): SM2022080-SM2022148

**Figure S7.** **Identification of *Salmonella* 1,4,[5],12:i:- isolates in this study (SM2022150-SM2023020).**

Upper row (Lanes 3-19): SM2022150-SM2022197

Lower row (Lanes 3-19): SM2022198-SM2023020

**Figure S8.** **Identification of *Salmonella* 1,4,[5],12:i:- isolates in this study (SM2023022-SM2023098).**

Upper row (Lanes 3-14): SM2023022-SM2023068

Lower row (Lanes 3-14): SM2023069-SM2023098

**Figure S9.** **Identification of *Salmonella* 1,4,[5],12:i:- isolates in this study (SM2023099-SM2023191).**

Upper row (Lanes 3-19): SM2023099-SM2023141

Lower row (Lanes 3-19): SM2023144-SM2023191

**Figure S10.** **Identification of *Salmonella* 1,4,[5],12:i:- isolates in this study (SM2023192-SM2023274).**

Upper row (Lanes 3-19): SM2023192-SM2023239

Lower row (Lanes 3-19): SM2023241-SM2023274

**Figure S11.** **Identification of *Salmonella* 1,4,[5],12:i:- isolates in this study (SM2023275-SM2023291).**

Lower row (Lanes 3-11): SM2023275-SM2023291

**Figure S12. Predicted coding capacity relative to the 3,002-locus cgMLST reference scheme across 281 *Salmonella* 1,4,[5],12:i:- ST34 isolates from Guizhou Province. (A)** Distribution of predicted protein-coding genes per genome (mean = 4,795.5; SD = 117.3; range 4,516-5,162). **(B)** Predicted protein-coding genes per genome by phylogenetic clade. For boxplots, the red horizontal line within each box denotes the median, the box bounds represent the interquartile range (IQR), and the whiskers extend to 1.5 × IQR. Individual isolates are overlaid as points. Clade 2 (n = 2) is shown as individual points only, without a box.
